# Updates to the checklist of nocturnal Macroheterocera (Lepidoptera) of the Central High Atlas of Morocco: One new species added for Morocco

**DOI:** 10.3897/BDJ.13.e137839

**Published:** 2025-01-29

**Authors:** Nidal Fetnassi, Omar Er-rguibi, Abdessamad Aglagane, Mohamed Ghamizi, Erki Õunap

**Affiliations:** 1 Department of Zoology, Institute of Ecology and Earth Sciences, Faculty of Science and Technology, University of Tartu, Tartu, Estonia Department of Zoology, Institute of Ecology and Earth Sciences, Faculty of Science and Technology, University of Tartu Tartu Estonia; 2 Laboratory of Water Biodiversity and Climate Changes (EauBiodiCc), Faculty of Sciences Semlalia, Cadi Ayyad University, Marrakech, Morocco Laboratory of Water Biodiversity and Climate Changes (EauBiodiCc), Faculty of Sciences Semlalia, Cadi Ayyad University Marrakech Morocco; 3 Research center of the Museum of Natural History of Marrakech, Cadi Ayyad University, Marrakech, Morocco Research center of the Museum of Natural History of Marrakech, Cadi Ayyad University Marrakech Morocco; 4 Higher Institute of Nursing Professions and Health Technics, Laayoune, Morocco Higher Institute of Nursing Professions and Health Technics Laayoune Morocco; 5 Laboratory of Biodiversity and Ecosystem Functioning, Faculty of Sciences, Ibn Zohr University, Agadir, Morocco Laboratory of Biodiversity and Ecosystem Functioning, Faculty of Sciences, Ibn Zohr University Agadir Morocco; 6 Institute of Agricultural and Environmental Sciences, Estonian University of Life Sciences, Tartu, Estonia Institute of Agricultural and Environmental Sciences, Estonian University of Life Sciences Tartu Estonia

**Keywords:** inventory, sugar bait trap, external morphology, genitalia dissection, DNA barcoding

## Abstract

**Background:**

This paper provides updates to the checklist of Macroheteroceran moths (Lepidoptera) of the central High Atlas of Morocco, following the initial inventory conducted by Charles Rungs nearly five decades ago. Sampling was carried out using sugar bait traps deployed across various habitat types in the region (natural, semi-natural and agricultural lands). Identification of the collected specimens involved a comprehensive approach, including examination of external morphology, dissections of genitalia and DNA barcoding.

**New information:**

In this study, we recorded a total of 123 species belonging to the families Noctuidae, Erebidae, Geometridae, Eutelidae and Drepanidae. *Euxoacos* (Noctuidae) was recorded as a new species for Morocco. The presence of *Apameamaroccana* (Noctuidae) and *Chersotisrungsi* (Noctuidae), both endemic to Morocco, was verified in the study area. Four of the 123 species were only identified at the genus level. Our inventory also sheds light on species that were previously not known to occur within our study area, reporting twelve species from the High Atlas Mountains for the first time. We also suggest omitting *Eupitheciafarinosa* (Geometridae) from the Moroccan Lepidoptera list. This study significantly contributes to uncovering an overlooked aspect of Lepidopteran biodiversity in Morocco, which is crucial for future conservation efforts.

## Introduction

The High Atlas Mountains, stretching across North Africa, stand as a region of remarkable biogeographic diversity and form a crucial part of the Mediterranean biodiversity hotspot (Médail and Quézel 2001, Myers 2003, Rankou et al. 2015). Characterised by its rich biodiversity and distinct ecosystems, the region plays a crucial role in the ecological balance, providing habitat for a diverse array of flora and fauna (El Alami et al. 2021, El Alami 2022). Situated between the warm, humid climate of the Mediterranean Sea to the north and the dry, arid conditions of the Sahara Desert to the south, the High Atlas Mountains serve as a unique climatic barrier (Funnell and Parish 1995). This juxtaposition of climates endows the region with diverse environmental conditions, making it an ideal habitat for a wide range of species, many of which are endemic to the region (Husemann et al. 2013).

Despite the well-documented diversity and ecological significance of the High Atlas Mountains, certain groups of animals, including many insects, have received disproportionately little attention. For example, the butterflies of Morocco have been extensively studied and the results have been summarised into a comprehensive catalogue by Tarrier and Declare (2008), with numerous subsequent revisions and updates being published (Tarrier and Andre 2016, Tarrier and Andre 2017, Tarrier 2018, Tarrier and Andre 2018). However, the exploration of nocturnal lepidopterans has not progressed at the same pace.

The most comprehensive catalogues of Moroccan Lepidoptera were compiled byRungs (1979) and Rungs (1981) nearly half a century ago. Since then, few studies have been published that report concise observations at specific localities during specific periods (e.g. Teobaldelli (1987), Mokhles (1989), Mokhles (1995), Mokhles (1996), Mérit (2014)). Although valuable at a wider scale, few subsequent updates (e.g. Koçak and Kemal (2009), Leraut (2009), Leraut (2019a), Leraut (2019b)) have not provided detailed information below the country level. Contemporary research involving Moroccan moths has primarily focused on narrowly limited groups, either within a taxonomic context (e.g. descriptions of new species and genera and taxonomic revisions of existing groups) (i.e. Hreblay (1994), Hausmann (1997), Hausmann et al. (2008), Kaila et al. (2019), Tabell et al. (2019)) or in applied sciences such as pest management (i.e. El Alaoui El Fels et al. (1999), El Alaoui El Fels et al. (2013), El Iraqui and Hmimina (2016), Sbay and Zas (2018), Boulamtat et al. (2021)).

Despite all these efforts, there is no comprehensive updated catalogue of Moroccan moths, with data on new species being scattered amongst various publications and, thus, difficult to follow. This paper aims to provide an update to the knowledge of Macroheteroceran fauna of the central High Atlas Mountains with the ultimate goal of enhancing the understanding of macromoth fauna of the region and establishing a basis for advancing research, conservation and management initiatives. With data being made public through the PlutoF database, we hope our discoveries are well accessible to future researchers.

## Materials and methods

### Study area

**Figure 1.**
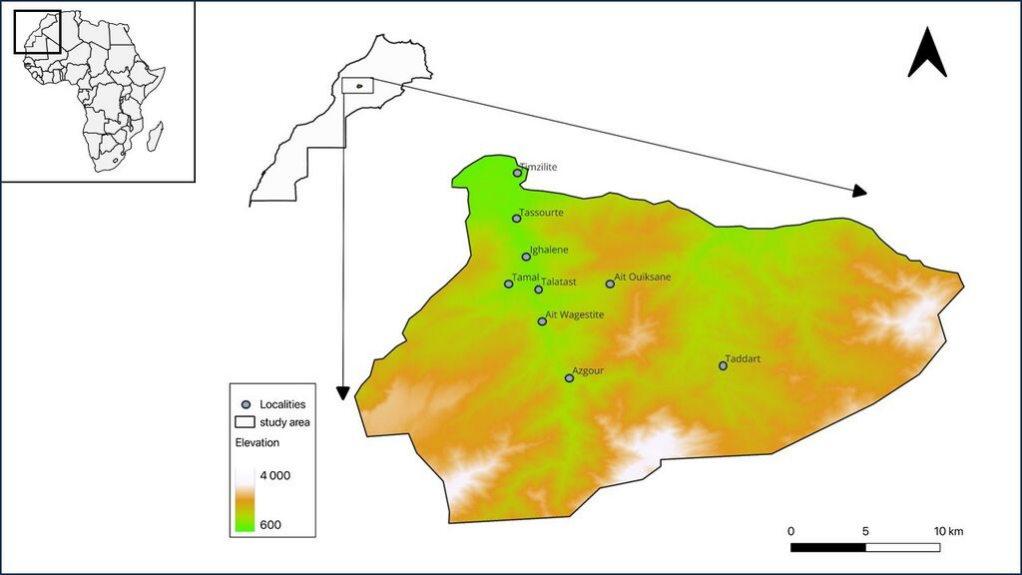
Map of the study localities.

**Figure 2.**
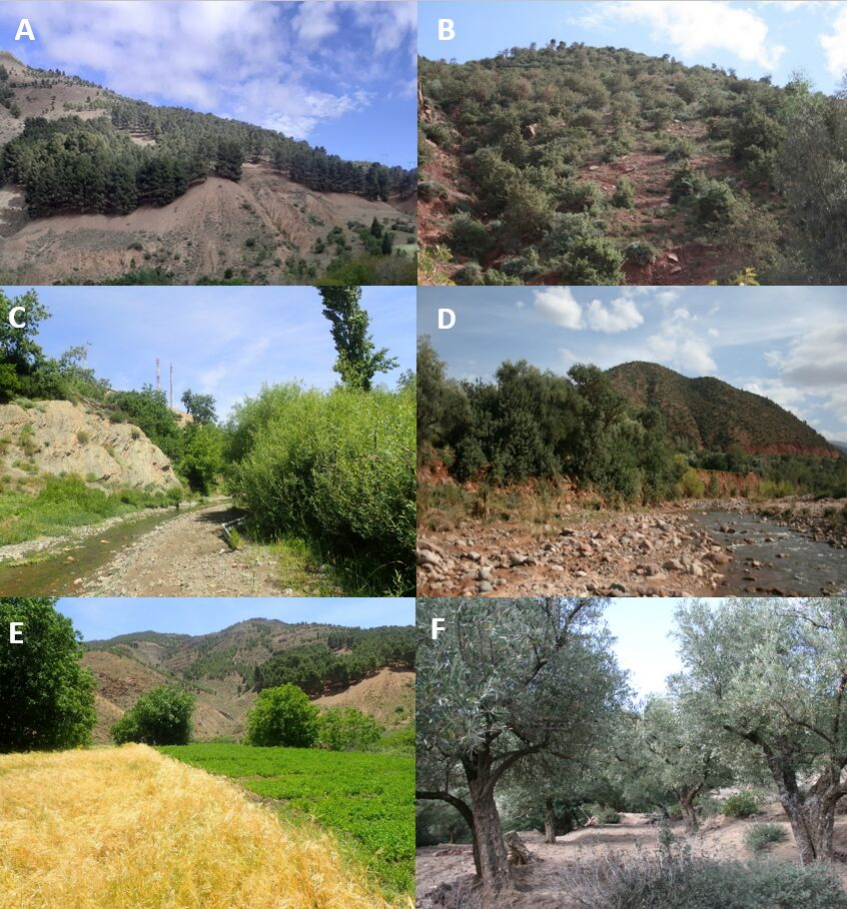
Various habitat types surveyed in the central High Atlas. **A** Natural habitat characterised by Aleppo pine forest in Taddart; **B** Natural habitat represented by heterogeneous forests in Tassourte; **C** and **D** Semi-natural habitat represented by river banks in Taddart and Timzilite, respectively; **E** Farmlands featuring annual crops in Taddart; **F** Farmlands with olive crops in Tamal. Photos by N. Fetnassi.

The study was conducted in the central High Atlas region of Morocco, covering nine villages (Fig. 1, Table 1). The High Atlas chain expands from the Atlantic margin of Morocco to the Mediterranean coast of Tunisia (Beauchamp et al. 1999). It is composed of faulted and folded Paleozoic, Mesozoic and Cenozoic rocks (Ayarza et al. 2005). The landscape of the study area is diverse, stretching from riverside lowlands and rugged cliffs to high peaks. The study area is characterised by a semi-arid climate, with two distinct seasons: a wet period from October to May and a dry period from June to September (Bouamri et al. 2018). Each locality within the overall study areas exhibits three distinct habitat components: natural forests, semi-natural vegetation (represented by river banks) and agricultural lands (Fig. 2). The forested areas were predominantly composed of *Juniperusphoenicea*, *J.oxycedrus* and *Quercusilex*, with certain localities also featuring *Pinushalepensis*. Riparian zones typically comprise *Populusalba*, *Rosacanina*, and *Salix* sp., with few areas additionally characterised by the presence of *Tamarixafricana* (Mostakim et al. 2022). Furthermore, agricultural fields were primarily dominated by crops of *Oleaeuropea*, though numerous regions also contained *Juglansregia* and a variety of annual crops.

### Identification of moths

Species identification was conducted using a tiered approach, emphasising both traditional morphological techniques and modern molecular methods to ensure accuracy, particularly given the complexity and relatively lesser-known status of African moths. Initially, an examination of external morphological characteristics, such as wing pattern, was performed, utilising observable features to distinguish between species. This foundational method, while effective for many taxa, occasionally proved inconclusive for certain specimens due to the subtle or overlapping nature of these external traits. In instances where morphological analysis alone was insufficient for definitive identification, dissection of the genitalia was undertaken to differentiate between closely-related or morphologically-similar species. We relied on a number of recent comprehensive publications (Fibiger 1990, Fibiger 1993, Fibiger 1997, Ronkay et al. 2001, Hacker et al. 2002, Goater et al. 2003, Fibiger and Hacker 2007, Fibiger et al. 2009, Leraut 2009, Zilli et al. 2009, Fibiger et al. 2010, Ronkay et al. 2011, Varga et al. 2013, Leraut 2019a, Leraut 2019b) to identify specimens based on their external morphology and genitalia dissection.

For dissecting genitalia, abdomens of moths were detached from the thorax and macerated for 20 h in a 15% potassium hydroxide (KOH) solution at room temperature before separating reproductive organs from the exoskeleton using a stereomicroscope (Leica s9i) (Leica Microsystems, Heerbrugg, Switzerland). For specimens that remained unidentified after studying their external and genital morphology, genomic DNA was extracted from one leg using the Qiagen DNeasy Blood and Tissue Kit following the manufacturer's protocol. A standard barcoding fraction (658 bp) of the mitochondrial COI gene (Hebert et al. 2004) was sequenced, following the protocol described in Õunap et al. (2021). The specimens are stored as voucher specimens in the Natural History Museum of University of Tartu and the Museum of Natural History of Marrakech. Habitus photographs were taken with a Canon EOS 700D DSLR camera, using a Canon EF 100 mm f/2.8L USM IS objective. Helicon Remote software was used for a series of multiple gradually focused images, which were thereafter stacked together using the Helicon Focus version 7.6.1 software. Genital slides were photographed with the built-in digital camera of the Leica S9i stereomicroscope. Images were cleaned and edited using Adobe Photoshop CS3 software.

### Sample collection

**Figure 3.**
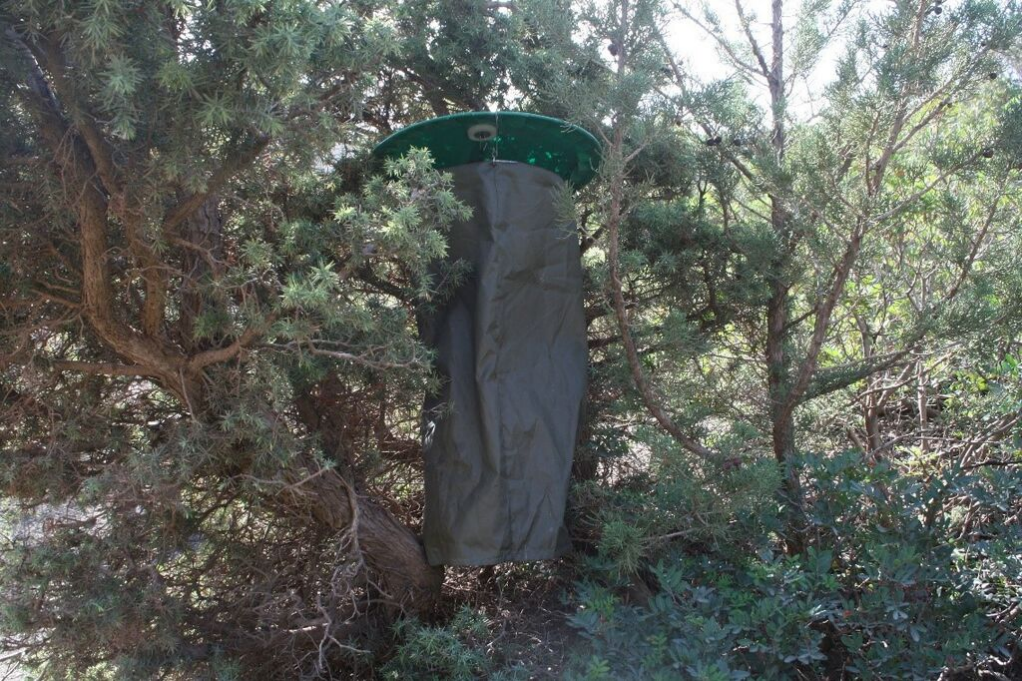
Sugar bait trap hanging in a natural habitat.

In this study, we used sugar bait traps (specifically the Jalas model, as described by Laaksonen et al. (2006)) (Fig. 3). These traps were primarily selected for logistical convenience, as they are easy to handle, unlike light traps, which require specialised equipment, along with constant monitoring and a reliable power source (Pettersson and Franzén 2008). Moreover, the green colour of the bait traps allows them to blend naturally into the environment, making them less conspicuous than light traps. Additionally, they can be left unattended for extended periods, with a maximum duration of one week in our case. Another key advantage of using bait traps is their ability to attract feeding species specifically (Süssenbach and Fiedler 1999, Freitas et al. 2014), which is particularly useful in regions where the moth fauna is not well known. This targeted approach simplifies species identification by narrowing the range of moths caught, in contrast to light traps.

The baits were composed of sponges soaked in a mixture of sugar and red wine placed within a 0.5-litre plastic cup, with ethyl acetate and chloroform serving as the lethal agents in the collecting jar. In 2021, the traps were set up overnight and checked the following day in Taddart. On the other hand, in other localities during 2022, details of trap operations varied depending on the season. During the dry season, the traps were checked every three days, whereas, in the wet season, the traps remained in place for 5 to 7 days before being checked. A total of 22 traps were deployed in Taddart, with sampling occurring over eight nights in 2021. These sessions were divided between early summer (13, 20 27 June and 4 July) and late summer (18 and 26 September and 2 and 9 October). In the rest of the localities, a total of 24 traps were set, with sampling spanning from 23 September to 15 November 2022, across 15 nights. Upon capture, the moths were placed in cotton envelopes for preservation, and individuals were either pinned or stored in a small entomological envelope to preserve the scales and to prevent any damage that might interfere with the identification process.

### Notes on the Checklist

The taxonomical order and nomenclature of the checklist follows Leraut (2009), Leraut (2019a), Leraut (2019b) and Top et al. (2023). This study presents illustrations of the habitus and genitalia for notable species, including those recently discovered in the High Atlas Mountains, newly identified in Morocco, endemic species and those with unresolved taxonomy at the genus level.

## Data resources

The species datasets that support our research are publicly accessible at https://dx.doi.org/10.15156/BIO/3059461 (Fetnassi 2024). These same datasets are also included in a larger dataset published on GBIF https://www.gbif.org/dataset/f1c4df18-12d6-40cb-ab51-5bb0d7f08d6e.

## Checklists

### Checklist of Macroheterocera species recorded in the central High Atlas of Morocco from late summer to early autumn 2021 and in autumn 2022

#### Watsonolla uncinula

(Borkhausen, 1790)

F2E40A0F-153A-5DDB-8D67-C6D597D11728

##### Notes

One specimen was collected from Azgour.

#### Rhodometra sacraria

(Linnaeus, 1767)

930BF00E-6563-5F6B-A1B4-37E16391EFD6

##### Notes

Two speciemens were collected for Taddart and Tamal.

#### Gymnoscelis rufifasciata

(Haworth, 1809)

F17D29D1-0FDE-57C1-8B52-FEF5B98277A0

##### Notes

27 specimens were collected from Taddart.

#### Eupithecia cooptata

Dietze, 1904

57A6D781-689A-5A63-BACC-0336C821C4CE

##### Notes

Two specimens were collected from Ait Ouiksane Village and subjected to barcoding. One male specimen, its genitalia and 8^th^ sternite are shown in Fig. 4. As summarised by Ratzel (2018), the identity of the Moroccan population of *E.cooptata* is still doubtful. He described a series of moths from High Atlas as E.cooptatassp.steineri Ratzel, 2018, but noted that its status is not sure and requires further study. Our two specimens were badly damaged in the bait trap and the abdomen was available for only one male. Genitalia dissection proved that this individual is, indeed, conspecific with *E.cooptatasteineri*. Interestingly, according to the barcodes, our Moroccan specimens are rather different (p-distance 0.0264) from the European sample (n = 1) of *E.cooptata* in the public data portal of Barcode of Life Data Systems (http://www.boldsystems.org/index.php) (referred to as BOLD hereinafter), thus supporting Ratzel’s view that this taxon may even be a distinct species. With just two damaged specimens in our possession, we could not perform a taxonomic rearrangement, but just stress the need for further study.

Ratzel (2018) noted that the type material of *E.cooptatasteineri* originated from a rather restricted area in the western part of the High Atlas Mountains. Our data increase the range of this taxon significantly, as Ait Ouiksane is located approximately 50 km southwest of the known localities of *E.cooptatasteineri*. Moreover, two more specimens in the BOLD (BC ZSM Lep 97868 from Igmir in the Anti Atlas and BC ZSM Lep 97891 from Ait Tamil in High Atlas) also have DNA barcodes identical or very similar (p-distance 0.0015) to our moths, suggesting that these specimens, currently identified as *E.farinosa* (Dietze, 1913), are actually also conspecific with *E.cooptatasteineri*, thus further increasing the known range of this species in Morocco.

As recently summarised by Ratzel (2018), *E.farinosa* is a form of *E.scopariata* (Rambur, 1833). It was described from Spain and, only subsequently, some African specimens have been associated with the name *E.farinosa* (for details, see Ratzel (2018)). As *E.scopariata* has exclusively European distribution (Mironov 2003, Leraut 2009) and is genetically very far (average p-distance 0.082 ± 0.001 SD) from our *E.cooptatasteineri* sample, it is obvious that individuals named as *E.farinosa* in the BOLD database have been misidentified. In conclusion, we suggest omitting *E.farinosa* from the list of Moroccan Lepidoptera.

#### Stegania trimaculata

(de Villers, 1789)

F5EE5029-A2DD-58DD-B58F-F8FB3E12C0AE

##### Notes

Two specimens were collected from Tamal.

#### Peribatodes powelli

(Oberthür, 1913)

1D44EC31-0625-5242-B9DA-12A6CC433B72

##### Notes

One specimen was collected from Ighalene.

#### Indalia uniola

(Rambur, 1866)

EF1AAACD-F7AC-5F44-B7B1-78FBAC4C91A3

##### Notes

One specimen was collected from Tamal.

#### Nodaria nodosalis

(Herrich-Schäffer, 1851)

C8BAE35C-23C6-5DAD-9EAC-5370F96B9E73

##### Notes

Four specimens were collected from Tamal.

#### Pechipogo simplicicornis

(Zerny, 1935)

D8871EE5-8A59-503F-9A48-DD771F08729C

##### Notes

One specimen was collected from Tamal.

#### Hypena obsitalis

(Hübner, 1813)

249DD73C-7972-5D76-923A-D74F2EE646EC

##### Notes

One specimen was collected from Timzilite.

#### Scoliopteryx libatrix

(Linnaeus, 1758)

4C7872A6-7108-5652-8B90-02C2390806B7

##### Notes

One male specimen was found in Taddart in the central High Atlas (Fig. 5a). According to Rungs (1981) and references therein, the species was not very abundant in Morocco. Rungs (1981) mentioned records from the Rif region, the Atlantic plains and low mountains (northeast and west) and the Middle Atlas, but no records from the High Atlas Mountains.

#### Schrankia costaestrigalis

(Stephens, 1834)

1C97D67C-4D1F-5385-886F-5C92590E93CC

##### Notes

One specimen was found in Azgour.

#### Parascotia nisseni

(Turati, 1905)

A4E4996F-DCF5-5E5A-8DA9-AA77EBABD45B

##### Notes

One specimen was collected from Taddart.

#### Antarchaea sp. (near erubescens)

11AB3FE9-0008-5EA7-B616-ABB464A51A01

##### Notes

We collected two specimens from Tamal and Tassourte. One adult male is illustrated in Fig. 5b. Rungs (1981) mentioned the genus *Phytometra* with three species: *P.vividaria*, *P.sanctiflorentis* and *P.erubescens*. Leraut (2019a) also briefly mentioned the presence of *P.erubescens* in North Africa. This ambiguity arises from our DNA barcoding results, which revealed a 97.33% match between our specimens and *P.erubescens*.

#### Eublemma ostrina

(Hübner, 1808)

8D518DD7-3619-537F-A451-CB87B9881F8D

##### Notes

One specimen was collected from Tamal.

#### Metachrostis velox

(Hübner, 1813)

B68323C9-ADB2-5BAD-8195-458B24CE6253

##### Notes

Four specimens were collected from Ait Ouiksane and Tamal.

#### Autophila cataphanes

(Hübner, 1813)

CE43015E-D3DC-51F3-8658-33F6F6B31DEB

##### Notes

Four specimens were collected from Azgour and Taddart.

#### Apopestes spectrum

(Esper, 1787)

FCE8990B-3822-5861-94B0-1D22B4453B75

##### Notes

One specimen was found in Azgour.

#### Catephia alchymista

(Denis & Schiffermüller, 1775)

B3CD9C07-B611-54F6-B75F-57F25B77027B

##### Notes

One specimen was collected from Ighalene.

#### Pandesma robusta

(Walker, 1858)

1AA873D0-68D2-5405-B2D2-B6BC722C7024

##### Notes

A total of 945 specimens were collected from all localities, except Talataste. The species is multivoltine, with polyphagous larvae that feed throughout the year. *P.robusta* is known to form permanent colonies, particularly in southern Europe, with a notable presence in Spain and the Andalusia Region. Its geographical distribution appears to be expanding, potentially due to the effects of global warming (Grange 2014).

#### Zethes insularis

Rambur, 1833

A7EC8571-CE2C-5C09-A7E6-7BFD280F2FD2

##### Notes

Seven specimens were collected from Ighalene, Taddart, Tassourte and Timzilite.

#### Heteropalpia acrosticta

(Püngeler, 1904)

A417FEA0-5BCF-5A83-A7A4-CF30830432D6

##### Notes

One specimen was collected from Azgour.

#### Catocala nymphaea

(Esper, 1787)

5036602D-AC7C-5796-8A29-2058564F4316

##### Notes

We recorded 1,701 specimens from Taddart. The species is known to feed on oaks (*Quercus* spp.) as larvae. Though no oaks were growing at our study site, a forest of green oak (*Quercusilex*) is located approximately 1 km away at an altitude of approximately 1700 m. Significant insect-driven damage to the foliage of oaks was detected there during our study period. Both *C.nymphaea* and *C.nymphagoga* have been considered as harmful defoliators in their larval stages, affecting various species of oaks in the Mediterranean Basin. This impact has been documented in several studies (Arahou 2008, Bouchaour-Djabeur 2013, Tiberi et al. 2016, Kacha et al. 2017).

#### Catocala nymphagoga

(Esper, 1787)

98CFA590-D5B6-57CA-8E27-5488DEF44400

##### Notes

Six specimens were collected from Taddart.

#### Catocala elocata

(Esper, 1787)

E98907D9-5D8C-5EC8-B8E0-D36F65BC0588

##### Notes

Five specimens were collected from Taddart. One male specimen is illustrated in Fig. 5c. Although Rungs (1981), summarising his own studies and earlier works by other researchers, had not documented *C.elocata* in the High Atlas Mountains, he speculated on its potential presence there. This species has previously been recorded in the Atlantic lowlands, low mountains (mainly in the north-western region of Morocco), the Souss Region and the Middle Atlas.

#### Catocala oberthuri

Austaut, 1879

2B6C6163-43C7-5A4F-B74F-796EA4D8C4E1

##### Notes

Ninety-one specimens were collected from most of our sampling sites of the Central High Atlas, except Talatast and Ait Ouagestite. One male specimen is illustrated in Fig. 5d. The species has previously been treated as a subspecies of *C.elocata* and was considered to be endemic to the Berber Region, even though some authors considered it as a different species at that time (Rungs 1981).

#### Catocala puerpera

(Giorna, 1791)

6BA733C8-93E6-511E-8709-7D31EACFD767

##### Notes

Seven specimens were collected from Azgour, Ighalene and Taddart.

#### Catocala dilecta

(Hübner, 1808)

266FB23E-0C7F-5755-A682-4DC32A30D156

##### Notes

Fourteen specimens were collected from Azgour, Ighalene and Taddart.

#### Catocala optata

(Godart, 1824)

60D66C2F-2CC5-5049-ABDF-F3E6CC45EB96

##### Notes

Twenty-one specimens were collected from Azgour, Ighalene, Taddart, Tassourte and Timzilite.

#### Tytroca dispar

(Püngeler, 1904)

D9E9C95E-8475-5962-91C7-4E7CF3E074E9

##### Notes

Seventy-four specimens were recorded from Ait Ouagestite, Ait Ouiksane, Ighalene, Taddart, Tamal, Tassourte and Timzilite.

#### Ophiusa tirhaca

(Cramer, 1777)

1F0001B9-F7BA-5D42-B093-B1D0E7FC2C34

##### Notes

One hundred and thirty-three specimens were collected from all our sampling localities, except for Ait Ouiksane.

#### Clytie illunaris

(Hübner, 1813)

7A353FB1-947A-5AFF-AAA9-39F32B680DED

##### Notes

One hundred and six specimens were collected from most of our sampling sites, except for Talatast and Ait Ouiksane. One female specimen is illustrated in Fig. 5e. Rungs (1981) noted that there had been limited instances where *C.illunaris* had been confused with *C.sancta* (Staudinger, 1898), suggesting that the references he cited for this species, Le Cerf (1924), Audeoud and Roch (1938) and Rungs (1938) might actually pertain to *C.sancta*, given that collectors often sampled in areas where *C.sancta* was abundant. Although Rungs (1981) did not personally encounter *C.illunaris* in the dissected material he examined, he posited that the species must inhabit areas where *Tamarixspp*. are present. Our specimens were verified through genitalia dissection and DNA barcoding, which provided a 99% match with *C.illunaris*.

#### Clytie infrequens

(Swinhoe, 1884)

06FFCD53-C7E5-5B5F-8558-FC296F9984A1

##### Notes

Only one male specimen was collected from Tamal (Fig. 5f). The species is known to occur in desert areas. Its distribution ranges from eastern Sahara through the Levant and the Arabian Peninsula to Pakistan and India (Katbeh-Bader 2017). Rungs (1981) had not previously reported the species from Morocco; however, subsequent works by Leraut (2019a) and Salem (2021) confirmed its presence across North Africa.

#### Dysgonia algira

(Linnaeus, 1767)

662F3D3A-A40B-57C8-8BCB-51A460210192

##### Notes

Twenty-three specimens were collected from Azgour, Ighalene, Taddart, Tamal and Timzilite.

#### Grammodes stolida

(Fabricius, 1775)

B57186E8-A3FC-57AB-97E8-C434B3BA8F25

##### Notes

Six specimens were collected from Timzilite.

#### Eutelia adulatrix

(Hübner, 1813)

FC10A7FB-9D01-5969-8328-B2EBFD99A3D1

##### Notes

Five specimens were collected from Tamal and Timzilite.

#### Thysanoplusia daubei

(Boisduval, 1840)

563E3103-550F-5458-BCAB-DE69CEA9EBB8

##### Notes

One specimen was collected from Timzilite.

#### Ctenoplusia accentifera

(Lefèbvre, 1827)

ECA880D2-F809-5B1E-A48E-D1ABD976748A

##### Notes

One specimen was collected from Ighalene.

#### Chrysodeixis chalcites

(Esper, 1789)

85058135-8D2B-5BF5-9CA0-2D492128C32D

##### Notes

Two specimens were collected from Tamal.

#### Autographa gamma

(Linnaeus, 1758)

2A0B1BCF-2F3E-5F63-B882-0D6542B3F2BE

##### Notes

One specimen was collected from Timzilite.

#### Acronicta psi

(Linnaeus, 1758)

9AE079CD-2A2D-53FC-A06C-4CDEC5EB7425

##### Notes

According to Rungs (1981), one caterpillar had been collected by Walker (1890) and, since then, the species had not been recorded in Morocco. Rungs (1981) also noted that the species bears a close resemblance to *A.radoti* (Le Cerf, 1924), which has been described from Morocco. According to the same author, there was a possibility that Walker had collected *A.radoti* instead of *A.psi*, given the uncertainty surrounding the occurrence of *A.psi* in Morocco during that period. Our specimens (n = 2) were collected from two localities, Ighalene and Timzilite and the DNA barcode showed a 99% match with *A.psi*. Habitus and female genitalia are illustrated in Fig. 6.

#### Acronicta rumicis

(Linnaeus, 1758)

DCD50C7C-0EAB-5E4B-9183-004B42369730

##### Notes

Eighty-eight specimens were recorded from all sampling localities, except for Ait Ouiksane.

#### Craniophora pontica

(Staudinger, 1879)

E8347DA3-A2E4-5E95-ADFD-222775C9FDE5

##### Notes

Fifteen specimens were collected from Taddart, Tamal, Ighalene and Timzilite. One female specimen is illustrated in Fig. 7a. According to Rungs (1981) and references therein, the species was occurring close to Rabat and in the Middle Atlas, but no records were known from the High Atlas Mountains.

#### Alvaradoia deserti

(Oberthür, 1918)

F83AC288-AAEA-5CC7-8ED5-AADA056CB017

##### Notes

One specimen was collected from Taddart.

#### Tyta luctuosa

(Denis & Schiffermüller, 1775)

18D1C463-D4C0-5273-8795-D4B4A0513378

##### Notes

One female specimen was collected from Timzilite (Fig. 7b). Rungs (1981) reported that this species occurs in northern Morocco, including the Rif Mountains and the Middle Atlas. The author noted that *T.luctuosa* had not been reported from the High Atlas Mountains, although its presence there seemed plausible. However, our findings confirm that the species does, indeed, inhabit the High Atlas Mountains.

#### Amphipyra tetra

(Fabricius, 1787)

D03C02FF-47AE-524A-B849-78D809EF06F0

##### Notes

Three specimens were collected from Taddart and Tamal.

#### Allophyes powelli

Rungs, 1952

C420445A-D5FF-59BE-BBF3-E2E3EC9CA643

##### Notes

Two specimens were collected from Talataste and Tassourte. According to Rungs (1981), the species was endemic to Morocco, but Ronkay et al. (2011) noted it is more widely distributed in the Maghreb countries.

#### Xylocampa mustapha

(Oberthür, 1910)

0AFDE4D5-DB37-5606-AD64-2897BD7FF110

##### Notes

One specimen was collected from Timzilite.

#### Heliothis peltigera

(Denis & Schiffermüller, 1775)

EEB166A3-4324-56E1-BACA-38DFE3B84372

##### Notes

Eighteen specimens were collected from Tamal, Tassourte and Timzilite.

#### Helicoverpa armigera

(Hübner, 1808)

6AE48AC5-20FC-5D56-906D-F5521504E244

##### Notes

Twenty-three specimens were collected from Ighalene, Taddart, Tamal and Timzilite.

#### Condica viscosa

(Freyer, 1831)

D8EC1CA2-26CB-5F5B-9532-2D8C762325B6

##### Notes

Three specimens were collected from Tamal and Timzilite.

#### Callopistria latreillei

(Duponchel, 1828)

D8B14C2F-ADFF-535C-AC54-6BE919CC170A

##### Notes

Eight specimens were collected from Azgour, Ighalene, Tamal, Tassourte and Timzilite.

#### Cryphia lusitanica

(Draudt, 1931)

EAD29781-E3CD-5DEA-9373-89424D353B1B

##### Notes

**Figure 8.**
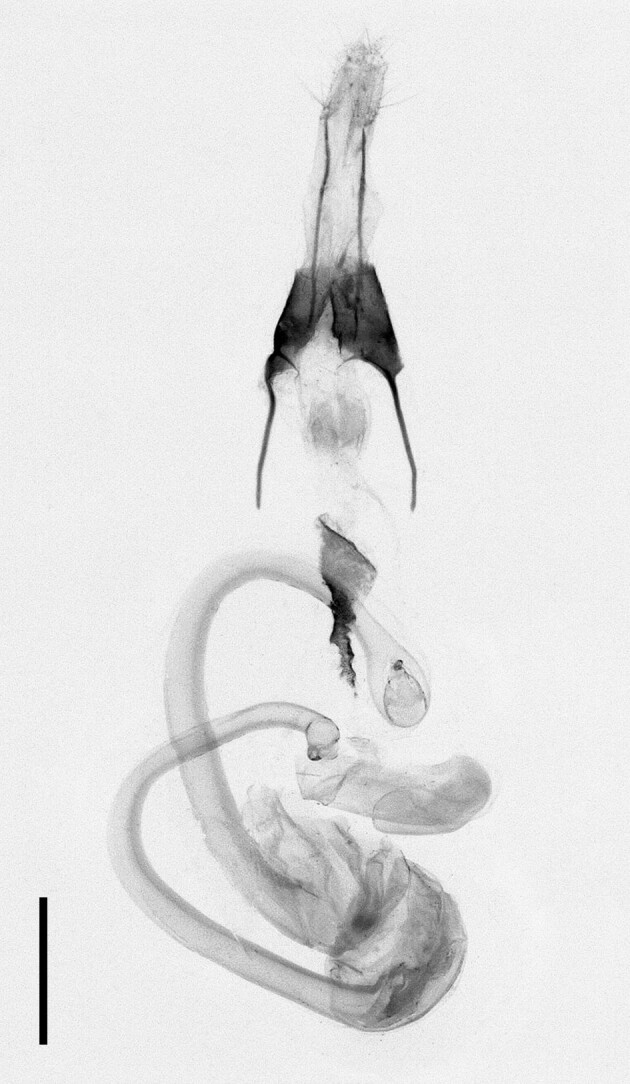
Genitalia of female *Cryphialusitanica* (Draudt) from High Atlas Mountains, taken at Tamal on 04/10/2022. Scale: 1 mm.

Two specimens were recorded from Tamal. For morphological confirmation, we hereby present the genitalia of one female *C.lusitanica* specimen (Fig. 8). The species occurs in the Iberian Peninsula, southern France and in the western extremity of Liguria in Italy (Govi and Fiumi 2019). Rungs (1981) had not previously reported the species from Morocco; however, subsequent works by Fibiger et al. (2009) confirmed its presence in northwest Africa.

#### Cryphia (Cryphia) sp. (near simulatricula)

4115F0A4-45BD-5BAE-913E-01C0136DAAA8

##### Notes

**Figure 9.**
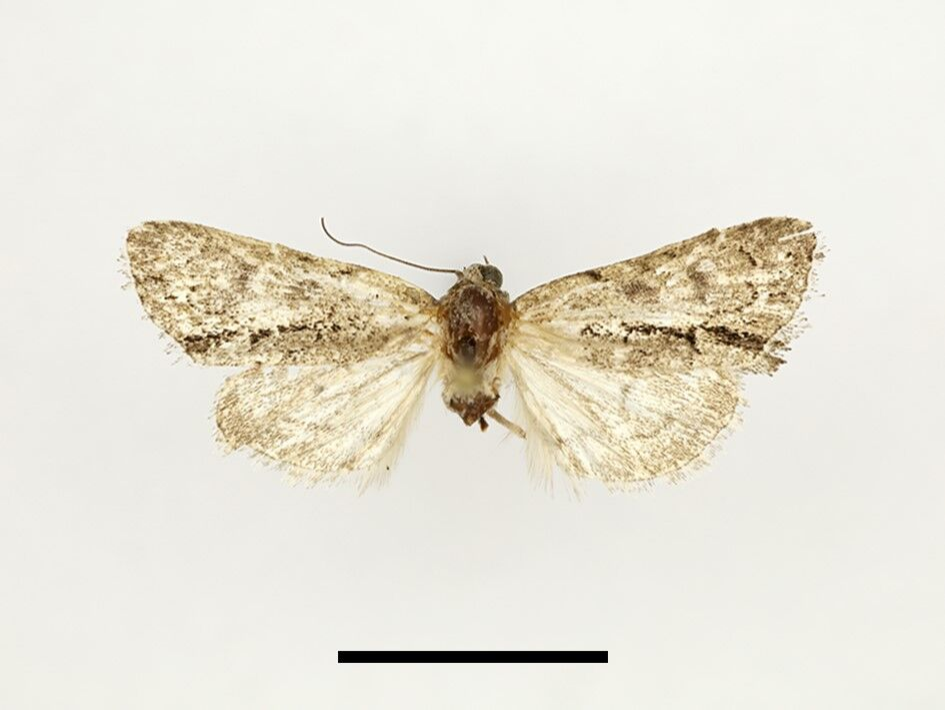
Female *Cryphia* sp (near simluatricula) from High Atlas Mountains, taken at Taddart on 18/09/2021. Scale: 1 cm.

One female specimen was collected from Taddart (Fig. 9). It had suffered significant damage in the trap, with part of the abdomen missing due to attacks by mantids. This left the specimen in a condition unsuitable for traditional identification methods, as critical genital structures were missing. Consequently, we resorted to DNA barcoding, which indicated a 100% match with *C.simulatricula* (Guenée, 1852). According to Fibiger et al. (2009) and Govi and Fiumi (2019), *C.simulatricula* is exclusively found in Europe, from south-eastern Spain across southern France to northwest Italy and southern Switzerland. Though the barcode of our specimen is identical to that of *C.simulatricula*, its external morphology is distinctly different, leaving its identity open.

#### Cryphia (Euthales) sp. (near pallida)

B8A22DAC-F647-520E-8EAB-99094D9F3DA5

##### Notes

We collected five females from Taddart, Tamal, Ighalene and Timzilite. One specimen, as well as its genitalia, are illustrated in Fig. 10. Upon dissection, these specimens exhibited similarities to a female of *C.pallida* (Bethune-Baker, 1894) as described in Noctuidae Europaeae vol. 11 (Fibiger et al. 2009). However, the results of DNA barcoding showed only a 97.8% to 98.16% match with this species, indicating a potential taxonomic discrepancy. Another potential identification for our specimens could be *C.rungsi* (Boursin, 1941), which has been described from Morocco. However, *C.rungsi* has never been barcoded and the morphology of its female genitalia is unknown (A. Zilli, *pers. comm.*)

#### Bryophila ravula

(Hübner, 1813)

78E6EBCF-525F-5CAA-A971-1637CEB5BD29

##### Notes

One specimen was collected from Taddart.

#### Spodoptera exigua

(Hübner, 1808)

98AEA4EF-3AF6-5AE5-8136-460E4C4859D8

##### Notes

Thirty-eight specimens were collected from Azgour, Ighalene, Taddart, Tamal and Timzilite.

#### Spodoptera cilium

Guenée, 1852

17718B0E-59A8-560D-AB44-A65C7C6FDD01

##### Notes

**Figure 11.**
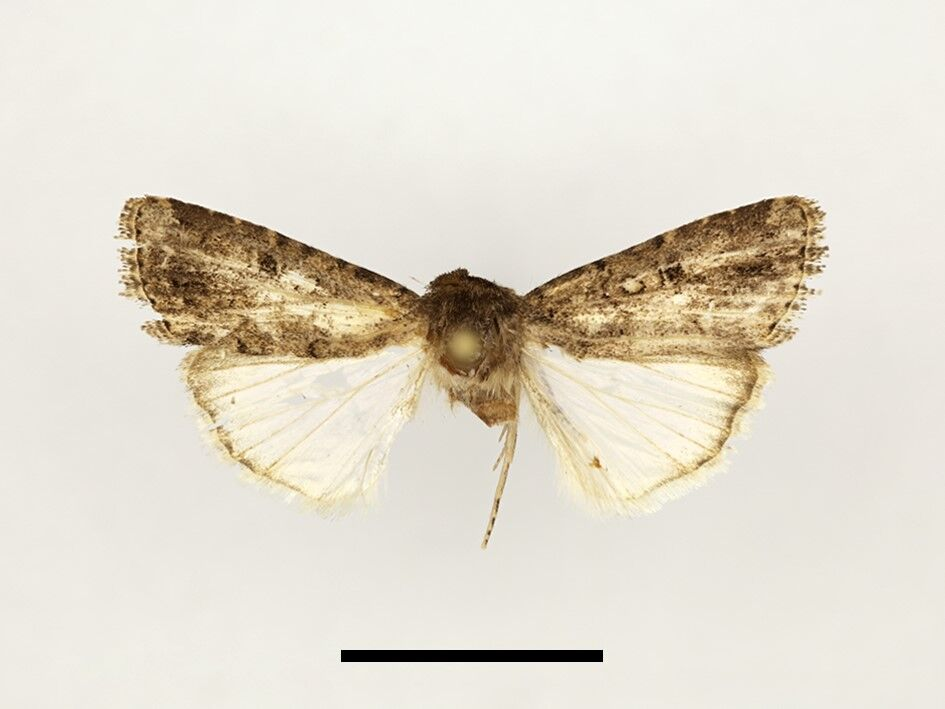
Male *Spodopteracilium* Guenée from High Atlas Mountains, taken at Timzilite on 04/10/2022. Scale: 1 cm.

Ten specimens were collected from Tassourte, Ighalene and Timzilite. One male specimen is illustrated in Fig. 11. According to Rungs (1981) and references therein, *S.cilium* has been recorded in the Middle Atlas, plains and low mountains, including Marrakech, which is considered to be the closest spot to our study localities. However, no earlier records are known from the High Atlas Mountains. The species is considered as a pest of various crops and can proliferate quite rapidly (Rungs 1981, Hatami et al. 2021), which might explain its vast expansion.

#### Spodoptera littoralis

(Boisduval, 1833)

E27AA0CE-C620-5EE1-AA99-8DBEDEE069F9

##### Notes

Eighty specimens were collected from Ighalene, Tassourte and Timzilite.

#### Caradrina proxima

Rambur, 1837

11D72343-F2F7-5332-93B2-66740728DDE3

##### Notes

Two specimens were collected from Timzilite.

#### Caradrina aspersa

Rambur, 1834

CBB6AC30-E4FE-5430-9048-B41479A5F7A7

##### Notes

Three specimens were collected from Ighalene and Timzilite.

#### Caradrina germainii

(Duponchel, 1835)

1B6CB0D1-0E8C-50BE-BD39-A1ED1922AD25

##### Notes

One specimen was collected from Timzilite.

#### Caradrina ingrata

Staudinger, 1897

997E0DFB-4C96-569B-B2F7-10F48934C975

##### Notes

One specimen was collected from Tamal.

#### Caradrina flava

Oberthür, 1876

E57D7CC5-72D5-5637-B3F1-2BE425B8F272

##### Notes

Four specimens were collected from Azgour, Ighalene and Taddart.

#### Caradrina sp. (near selini)

CB5B61BF-C53B-5B8E-9633-481FE72699A3

##### Notes

We collected two specimens from Azgour. As wings of both specimens were heavily damaged in the trap, we hereby present illustrations of their genitalia in Fig. 12. Upon dissecting the specimens, similarities were noted with both *C.flavirena* and *C.noctivaga*. However, the wings of the moths were significantly damaged, preventing detailed study of their external morphology. Subsequent DNA barcoding of these specimens revealed a 97.24% match with *C.selini*, with *C.flavirena* and *C.noctivaga* being approximately as distant from our specimens (97.14% and 96.7%, respectively).

#### Caradrina flavirena

Guenée, 1852

EF58633C-9C9D-55E7-AC32-B5F173F181FF

##### Notes

Thirty-nine specimens were collected from five localities in Central High Atlas: Azgour, Tamal, Ighalene, Timzilite and Ait Ouagestite. We provide illustrations of an adult female (Fig. 13a) and a male genitalia (Fig. 13b). The species has previously been mentioned from the Atlantic plains and low mountains, from the Middle Atlas as well as the Tell Atlas, but no records are known from the High Atlas (Rungs 1981).

#### Caradrina noctivaga

Bellier, 1863

C8792F62-4376-5942-8B04-AF006729FB52

##### Notes

Four specimens were collected from Tamal and Timzilite.

#### Caradrina clavipalpis

(Scopoli, 1763)

11DECEE4-D536-5508-AAED-4B634492ABF1

##### Notes

Fourteen specimens were collected from Ighalene, Taddart, Tamal, Tassourte and Timzilite.

#### Hoplodrina ambigua

(Denis & Schiffermüller, 1775)

F50AA2E1-069C-5BFA-A070-774A40E8DE07

##### Notes

Six specimens were collected from Tassourte and Timzilite.

#### Anthracia ephialtes

(Hübner, 1822)

B2DB52B2-E306-5E57-9097-CDBF3F9770E1

##### Notes

Ten specimens were collected from Taddart, Tamal and Timzilite.

#### Mormo maura

(Linnaeus, 1758)

78399CF3-D26F-55C9-8C7A-CAC981E2F249

##### Notes

Forty-five specimens were collected from Azgour, Ighalene, Taddart, Tamal and Timzilite.

#### Olivenebula xanthochloris

(Boisduval, 1840)

FF5D64A5-35DD-51B1-815B-56437B1DDF93

##### Notes

Four specimens were collected from Taddart.

#### Thalpophila vitalba

(Freyer, 1834)

80A45EB7-2B2A-588F-9281-882632B65908

##### Notes

Two specimens were collected from Taddart.

#### Pseudenargia ulicis

(Staudinger, 1859)

E659C225-304B-55F3-9D1B-8F0A67FDE20F

##### Notes

Twenty specimens were collected from Azgour, Ighalene and Taddart.

#### Apamea maroccana

(Zerny, 1934)

0FACA0FF-7D6E-5DC2-8090-4A6616F25A36

##### Notes

**Figure 14.**
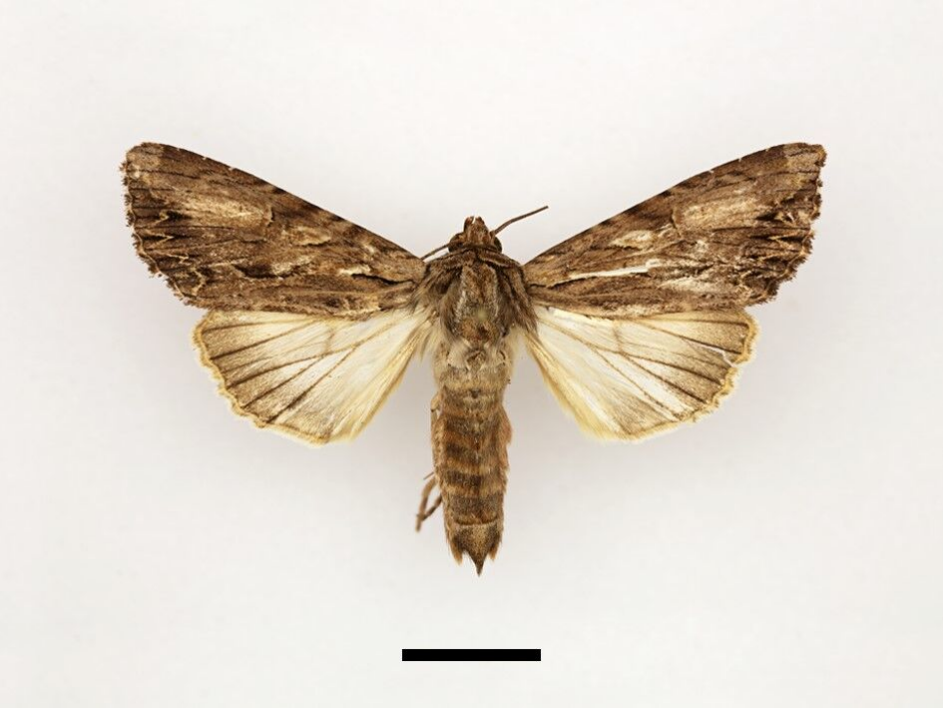
Male *Apameamaroccana* Zerny from High Atlas Mountains, taken at Taddart on 13/06/2021. Scale: 1 cm.

Four specimens were collected from Taddart. One male specimen is illustrated in Fig. 14. According to Rungs (1981) and Zilli et al. (2009), the species is endemic to Morocco.

#### Atethmia algirica

(Culot, 1914)

2CB1D003-D1C5-5AB0-A38E-2353A5CEF83F

##### Notes

One specimen was collected from Taddart.

#### Agrochola lychnidis

(Denis & Schiffermüller, 1775)

8400B715-6309-56E1-A30B-8AE6EA89E573

##### Notes

Four specimens were collected from Azgour, Ighalene and Tamal.

#### Anchoscelis meridionalis

(Staudinger, 1871)

8F01952D-6BAE-5E1D-848A-3D1264D8D03D

##### Notes

Two specimens were collected from Azgour.

#### Dryobota labecula

(Esper, 1788)

824FBC31-E0EA-542B-92B0-F445B5ADE8EA

##### Notes

Seven specimens were collected from Azgour.

#### Dryobotodes eremita

(Fabricius, 1775)

0A11898B-EEF7-5C6D-BE2B-02270AE9591A

##### Notes

Three specimens were collected from Azgour, Taddart and Tassourte.

#### Dryobotodes monochroma

(Esper, 1790)

3531FB7B-4E11-5B5E-8BEE-F8200405B0C9

##### Notes

Thirty-five specimens were collected from most of the localities, except for Ait Ouiksane and Talataste.

#### Dryobotodes roboris

(Geyer, 1835)

FA0829D0-AAAC-50FE-B2C9-3A58728CF8A6

##### Notes

One specimen was collected from Azgour.

#### Dryobotodes tenebrosa

(Esper, 1789)

7C2ADAC2-3EED-5E26-AD8C-C9645BF1D9C1

##### Notes

Seven specimens were collected from Ighalene, Taddart and Tamal.

#### Ammopolia witzenmanni

(Standfuss, 1890)

79710CFE-092D-57E9-A4FF-028F9375D647

##### Notes

Ten specimens were collected from Ighalene, Taddart and Tamal.

#### Trigonophora flammea

(Esper, 1785)

8C944ECD-1A7C-58DF-99FA-8037B78ACD65

##### Notes

Nineteen specimens were collected from Azgour, Ighalene, Tamal and Timzilite.

#### Trigonophora crassicornis

(Oberthür, 1918)

A2DE4881-4037-5172-A6D9-E46458CABC29

##### Notes

Two specimens were collected from Azgour.

#### Aporophyla nigra

(Haworth, 1809)

49C3AF16-E674-507E-B8F2-65E4CA8A14A8

##### Notes

One specimen was collected from Tamal.

#### Polymixis lichenea

(Hübner, 1813)

33F3F56B-7B35-5730-943F-B694A872E976

##### Notes

Four specimens were collected from Taddart.

#### Polymixis xanthomista

(Hübner, 1819)

1EF5D45B-D01D-5E16-875A-BE4358EE101B

##### Notes

Three specimens were collected from Azgour.

#### Polymixis flavicincta

(Denis & Schiffermüller, 1775)

2BDA0008-9EDB-5FA2-A28B-0B58E3C506DC

##### Notes

Twelve specimens from Azgour, Ighalene and Tamal.

#### Polymixis germana

(Rothschild, 1914)

0D4EA722-23A4-5C45-BD4A-542A06E007DA

##### Notes

Seven specimens were collected from Ait Ouagestite, Ighalene and Tamal.

#### Polymixis subvenusta

(Püngeler, 1906)

6D4C6495-5244-5BDC-9760-4A3BAC00EF3C

##### Notes

We collected seven specimens from Azgour, Taddart and Tamal. We provide illustrations of a male habitus in Fig. 15a and male genitalia in Fig. 15b. The genitalia of the dissected specimens did not correspond to any *Polymixis* species documented in literature used for this study. Thus, five speciemens were subjected to DNA barcoding, which revealed close to 2% genetic distance within the sample. As some of the studied individuals had barcode almost identical (99.69% match) to that of a *P.subvenusta* specimen presented in the BOLD database and genitalia of genetically most distant specimens were identical, we concluded that our material belongs to that species. *P.subvenusta* has been reported from the High Atlas Mountains by Rungs (1981).

#### Mniotype occidentalis

Yela, Fibiger, Ronkay & Zilli, 2010

D7A3BC77-C356-57BA-9CF4-CF71DEC63AE7

##### Notes

Thirty-two specimens were collected from Tassourte, Azgour, Tamal, Ighalene and Timzilite. One male specimen is illustrated in Fig. 16a. Its distribution ranges from northwest Africa across the Iberian Peninsula to southwest France (Fibiger et al. 2010). Rungs (1981) reported only *M.spinosa* from Morocco, referring to it as *Blepharitaspinosa* (Chrétien, 1910), which he considered to be synonymous with *B.solieri* (Boisduval, 1829). Subsequent studies recognised *M.occidentalis* as a distinct species within the traditional concept of *M.spinosa* from Continental Europe and Morocco. These species are morphologically very similar, with the main distinguishing feature being the presence or absence of the abdominal brush organs (TBO) in males. Though notably weak, these organs are present in both *M.spinosa* and *M.solieri*, but are lacking in *M.occidentalis*, as indicated by Fibiger et al. (2010). According to the same authors, *M.spinosa* is present outside Europe in northeast Africa, specifically in Algeria and Tunisia. We did not detect TBO in any of the dissected specimens; thus, our specimens can be confirmed as *M.occidentalis*.

#### Anarta trifolii

(Hufnagel, 1766)

0D810A9D-9BFB-5AA1-B51B-91694567750E

##### Notes

Nine specimens were collected from Ighalene, Tamal and Timzilite.

#### Hecatera dysodea

(Denis & Schiffermüller, 1775)

5E657679-B6D3-5F3A-84F2-1199831E0724

##### Notes

One specimen was collected from Azgour.

#### Mythimna vitellina

(Hübner, 1808)

01682CEF-846E-5A9D-9AD6-181187AA077C

##### Notes

Four specimens were collected from Taddart and Tamal.

#### Mythimna unipuncta

(Haworth, 1809)

2E1DA1FF-0640-5AF2-A8DF-D18446F01BBC

##### Notes

Eighty-eight specimens were collected from Ighalene, Taddart, Tamal, Tassourte and Timzilite.

#### Mythimna languida

(Walker, 1858)

443F2FAA-DDD5-5B88-9893-203A61D4643E

##### Notes

Ten specimens were collected from Tassourte, Tamal, Timzilite and Ighalene. One female specimen is illustrated in Fig. 16b. The species is notably recognised for its rapid expansion northwards and westwards in the western Palearctic Region due to global warming (Yela and De Vrieze 2002). It is regarded as an occasional migrant in Europe (Leraut 2019b), with numerous records across the continent, particularly from the Islands of Corsica, Sicily, Crete, Corsica and Cyprus, southern Italy and Greece, the Balkans, Germany and Macedonia (Rezbanyai-Reser and Hausmann 2000a, Rezbanyai-Reser and Hausmann 2000b). In Africa, the occurrence of *M.languida* has been documented in several countries, but there are no published records from Morocco (Rezbanyai-Reser and Hausmann 2000a, Rezbanyai-Reser and Hausmann 2000b, Yela and De Vrieze 2002, Leraut 2019b).

#### Mythimna albipuncta

(Denis & Schiffermüller, 1775)

DF0FB9BE-82C2-54F3-ABF8-89B355463F2F

##### Notes

Six specimens were collected from Taddart and Timzilite.

#### Mythimna congrua

(Hübner, 1817)

0D7E763D-3F2A-5444-8F58-0869183ED08E

##### Notes

Only one female specimen was collected from Taddart (Fig. 16c). This species was not included in the catalogue of Rungs (1981), but subsequently, both Hacker et al. (2002) and Leraut (2019b) have stated that *M.congrua* is Holomediterranean, exhibiting distribution across the Mediterranean Basin, from Morocco to southern Europe (Spain, Italy and Greece), including the islands of Corsica, Sardinia, Sicily and Crete, as well as western Asia.

#### Mythimna algirica

(Oberthür, 1918)

AEBAFF3F-5153-5DDA-8CEB-C16C791DFCF7

##### Notes

Six specimens were recorded from Ighalene, Taddart and Timzilite.

#### Mythimna l-album

(Linnaeus, 1767)

ECDF4000-CC51-5B57-8D21-2E209B4313F2

##### Notes

Fifteen specimens were collected from Ighalene, Taddart, Tamal, Tassourte and Timzilite.

#### Leucania zeae

(Duponchel, 1828)

10F94D0F-103C-565B-9276-D065062E6131

##### Notes

Only one male specimen was collected from Timzilite (Fig. 16d). Previous records of *L.zeae* are known from northeast and west Morocco, the Middle Atlas, as well as the southeast of Morocco in Erfoud, which is located in the desert area, but no records are known from the High Atlas (Rungs 1981).

#### Leucania putrescens

(Hübner, 1824)

58F308B9-5FA9-5E4F-BF1C-B15DA1F831B1

##### Notes

Ninety-two specimens were collected from most of the localities, except for Ait Ouagestite, Azgour and Talataste.

#### Leucania punctosa

(Treitschke, 1825)

CD70D68F-B0C9-5952-9217-747B9CDCA248

##### Notes

Only one specimen was collected from Timzilite.

#### Leucania loreyi

(Duponchel, 1827)

CCCD3B5C-7062-5020-9A31-58A723BE3419

##### Notes

Two specimens were collected from Timzilite.

#### Peridroma saucia

(Hübner, 1808)

3A618669-970A-525B-940C-38A1F0528BF7

##### Notes

Eleven specimens were collected from Taddart, Tamal and Timzilite.

#### Dichagyris flammatra

(Denis & Schiffermüller, 1775)

CC593D4C-247C-53A1-880D-B0192E8A08FE

##### Notes

Five specimens were collected from Taddart, Tamal, Tassourte and Timzilite.

#### Dichagyris constanti

(Millière, 1860)

8ADD4C00-A539-5740-A42D-A6E8AECC1B95

##### Notes

Eight specimens were collected from Ait Ouiksane, Azgour, Taddart, Talataste and Tassourte.

#### Euxoa temera

(Hübner, 1808)

A437FFCD-6BA5-5BFB-BC44-EE33849D0152

##### Notes

Seven specimens were collected from Tamal, Ait Ouagestite and Ait Ouiksane. One male specimen is illustrated in Fig. 16e. This species has previously been recorded only from the Middle and the Tell Atlas, but no records are known from the High Atlas (Rungs 1981).

#### Euxoa obelisca

(Denis & Schiffermüller, 1775)

AD0AAD69-0EFC-5B5B-989F-A8E840C512BB

##### Notes

Two specimens were collected from Taddart.

#### Euxoa cos

(Hübner, 1824)

866237D7-8C15-546C-8986-D108F69DC754

##### Notes

Only one heavily-damaged (metathorax and abdomen missing) female specimen was collected from Taddart (Fig. 16f). The identification is based on DNA barcoding (99.7% match) and the wing pattern of the forewings is also congruent with that of *E.cos*. The species is known to occur in southern Europe. However, outside Europe, there have been records from Algeria, TunisiaT and the Middle East (Fibiger 1990, Leraut 2019b), but no records from Morocco. All studies available on the Zoological Records database on Moroccan moths reveal no prior reports of this species. Additionally, we are unaware if these species are held in any private collections, as there have been no reports confirming this. Consequently, based on this lack of earlier data, we conclude that this record represents a new species for Morocco.

#### Euxoa hastifera

(Donzel, 1847)

82EF45CB-6AF8-503C-BE1B-4B0C1C57CF9F

##### Notes

Two specimens were collected from Tamal.

#### Agrotis segetum

(Denis & Schiffermüller, 1775)

44E2C0A9-2D28-5F5F-B47D-0A76E93F6DD5

##### Notes

One hundred and seventeen specimens were collected from Azgour, Ighalene, Taddart, Tamal, Tassourte and Timzilite.

#### Agrotis trux

(Hübner, 1824)

21E3265E-3160-5D67-9F9A-96BAC0FEB11D

##### Notes

Seventy specimens were collected from most of the localities, except for Ait Ouagestite and Ait Ouiksane.

#### Agrotis ipsilon

(Hufnagel, 1766)

F63A371A-4822-53B8-9568-040C421A96A7

##### Notes

Twenty-four specimens were collected from most of the localities, except for Ait Ouagestite and Talatatse.

#### Agrotis spinifera

(Hübner, 1808)

07AE85C8-FDF3-5C46-8D74-92DF584A84E1

##### Notes

Seven specimens were collected from Taddart, Tassourte and Timzilite.

#### Ochropleura leucogaster

(Freyer, 1831)

5859D237-04A5-50B2-BBF4-181A4BB53BD5

##### Notes

Three specimens were collected from two localities, Taddart and Timzilite. One female specimen is illustrated in Fig. 17a. The species has previously been recorded from the Rif Mountains, the Atlantic plains from Tangier to Casablanca and the Middle Atlas, but no records are known from the High Atlas (Rungs 1981).

#### Chersotis rungsi

Boursin, 1944

2E8B03FE-9C76-578D-8D0E-86A321DEF2EE

##### Notes

Two specimens were collected from Taddart. One male specimen is illustrated in Fig. 17b. According to Rungs (1981) and Varga et al. (2013), the species is endemic to Morocco.

#### Noctua comes

Hübner, 1813

0F98F34F-2F8E-543B-BAF2-4F6E8DED9135

##### Notes

Thirty-one specimens were collected from most of the localities, except for Ait Ouagestite and Talataste.

#### Xestia kermesina

(Mabille, 1869)

BCB610ED-A0C5-5AF0-B8A9-2A8B73703A30

##### Notes

Seventy-eight specimens were collected from most of the localities, except for Ait Ouagestite and Talataste.

#### Xestia xanthographa

(Denis & Schiffermüller, 1775)

BDEF7679-0C5A-5D57-9976-F30B4297DA9F

##### Notes

Twenty specimens were collected from Ighalene, Taddart and Timzilite.

#### Xestia c-nigrum

(Linnaeus, 1758)

66649CAE-B5F5-593A-B1AF-D142C5A4DFE1

##### Notes

One hundred specimens were collected from Ighalene, Taddart, Tamal, Tassourte and Timzilite.

## Discussion

We collected 4553 specimens of Macroheterocera representing 123 species from the families Noctuidae, Erebidae, Geometridae, Drepanidae and Eutelidae. Amongst these, two species are endemic to Morocco, as indicated by Rungs (1981): *Apameamaroccana* (Zerny, 1934) and *Chersotisrungsi* Boursin, 1944. The majority of the species, accounting for 70%, belong to the family Noctuidae, followed by 23% in Erebidae, 4% in Geometridae and less than 1% each in both Eutelidae and Drepanidae. The disparity in the proportions of recorded species can be attributed to the different feeding habits of various moth families. Many species belonging to the families Noctuidae and Erebidae are fruit-feeders as adults (Süssenbach and Fiedler 1999, Freitas et al. 2014). In contrast, most adult Geometridae and Drepanidae species primarily feed on flowers. This behavioural distinction is linked to the physical characteristics of these families: noctuids and erebids are typically more robust, featuring a proportionately large thorax and smaller wings. In contrast, geometrids are more slender and are characterised by a smaller thorax and larger wings. These structural differences are likely associated with varying energy requirements, as the more robust noctuids and erebids have higher energy demands than their slender geometrid counterparts, which may explain their higher representation amongst the recorded species (Utrio 1983). Notably, 70% of the specimens are from the family Erebidae, while 29% are from Noctuidae and less than 1% from other families. This unusually high proportion of Erebidae specimens derives from a significant outbreak involving two species, *Catocalanymphaea* (Esper, 1787) and *Pandesmarobusta* (Walker, 1858). Additionally, there may be potential bias introduced by the bait-trapping technique. According to Süssenbach and Fiedler (1999), while this method can influence sample size, it does not affect the estimates of community structure derived from the samples.

By comparing our results with the references available on the "Zoological Records" database, we recorded one species as new for Morocco: *Euxoacos* (Hübner, 1824). Compared to the study by Rungs (1981), which has been the only national reference on Moroccan Lepidoptera, we found twelve species as new to our study area, namely *Scoliopteryxlibatrix* (Linnaeus, 1758), *Catocalaelocata* (Esper, 1787), *Catocalaoberthuri* Austaut, 1879, *Clytieillunaris* (Hübner, 1813), *Acronictapsi* (Linnaeus, 1758), *Craniophorapontica* (Staudinger, 1879), *Tytaluctuosa* (Denis & Schiffermüller, 1775), *Spodopteracilium* Guenée, 1852, *Caradrinaflavirena* Guenée, 1852, *Leucaniazeae* (Duponchel, 1828), *Euxoatemera* (Hübner, 1808) and *Ochropleuraleucogaster* (Freyer, 1831).

Additionally, our study revealed six species that had not been reported from Morocco by Rungs (1981), but were later recorded by other foreign specialists; these are *Eupitheciacooptata* Dietze, 1904, *Clytieinfrequens* (Swinhoe, 1884), *Cryphialusitanica* (Draudt, 1931), *Mniotypeoccidentalis* Yela, Fibiger, Ronkay & Zilli, 2010, *Mythimnalanguida* (Walker, 1858) and *Mythimnacongrua* (Hübner, 1817). Despite using the three identification methods (external morphology examination, genitalia dissection and molecular barcoding), we could not identify four species that remained at the genus level. These include *Antarchaea* sp. (near erubscens), *Cryphia* sp. (near simulatricula), *Cryphia* sp. (near pallida) and *Caradrina* sp. (near selini).

Our findings highlight significant implications for understanding species distribution dynamics. Our results suggest two plausible scenarios for the presence of species that were not recorded by Rungs (1981). Firstly, it is possible that these species were overlooked in previous studies due to their rarity, which could have made them less visible or totally absent during sampling efforts. On the other hand, the existence of these species in our study area could testify to their recent introduction or to an expansion of their distribution within Morocco. This postulate suggests that these species may have been absent from the region during previous studies and have since become established. The reasons for this expansion remain enigmatic, yet they may be associated with ecological shifts and climate change (Warren et al. 2001, Hällfors et al. 2023).

In conclusion, nocturnal Lepidoptera are frequently neglected in certain areas, making it difficult to gather accurate and representative data. As a result, understudied species may be overlooked in monitoring and conservation initiatives, potentially jeopardising efforts to protect and conserve this group. This report represents an update to the checklist of Macroheterocera within the Central High Atlas of Morocco. Through meticulous data collection and analysis, we recorded 123 species thriving across various habitats in the area. Amongst these, two species were known endemics of Morocco and one species was newly reported from Morocco. For twelve species, at least one locality was known from Morocco before our study, but their presence in the High Atlas Mountains had never been reported. Another six species had been reported from Morocco after the major catalogue of Rungs (1981) by other authors (e.g. Hacker et al. (2002), Fibiger et al. (2010), Leraut (2019b)), but without specific data on collecting localities, thus leaving their presence or absence in the High Atlas Mountains open. This work also marks the beginning of a more in-depth study of the lepidopterological fauna in Morocco, with the intention of expanding our knowledge and continuing the documentation of these fascinating species in Morocco in the years to come.

## Figures and Tables

**Figure 4a.**
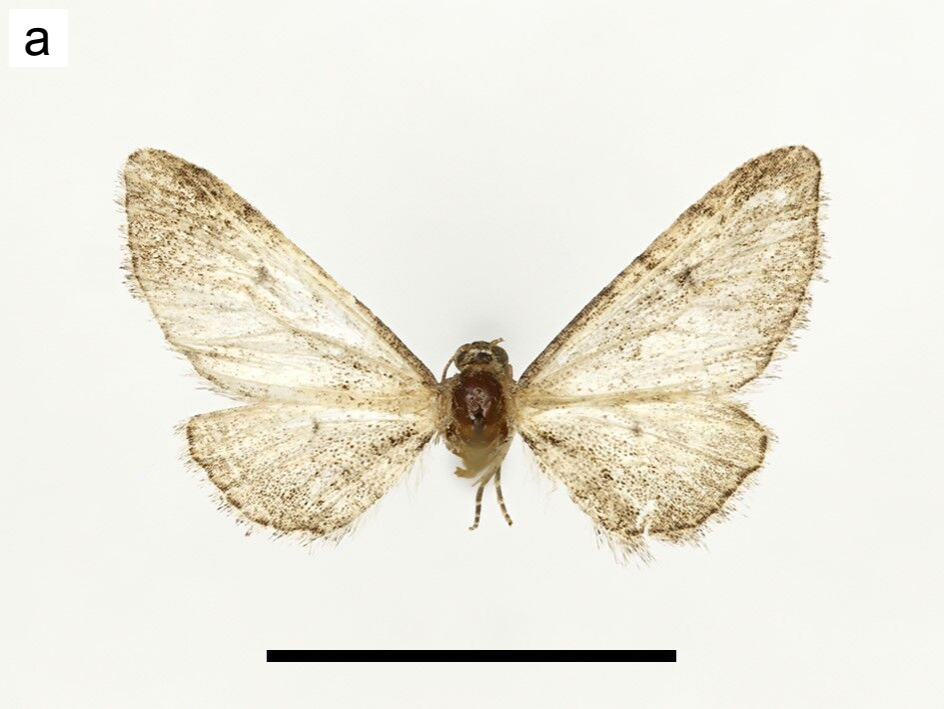
Habitus. Scale: 1 cm;

**Figure 4b.**
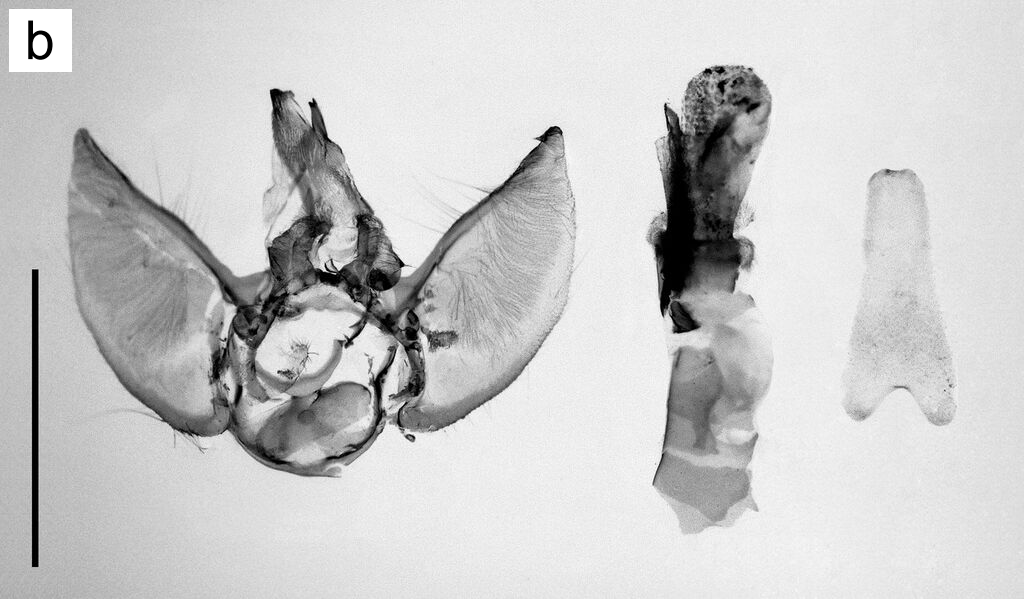
Genitalia. Scale: 1 mm.

**Figure 5a.**
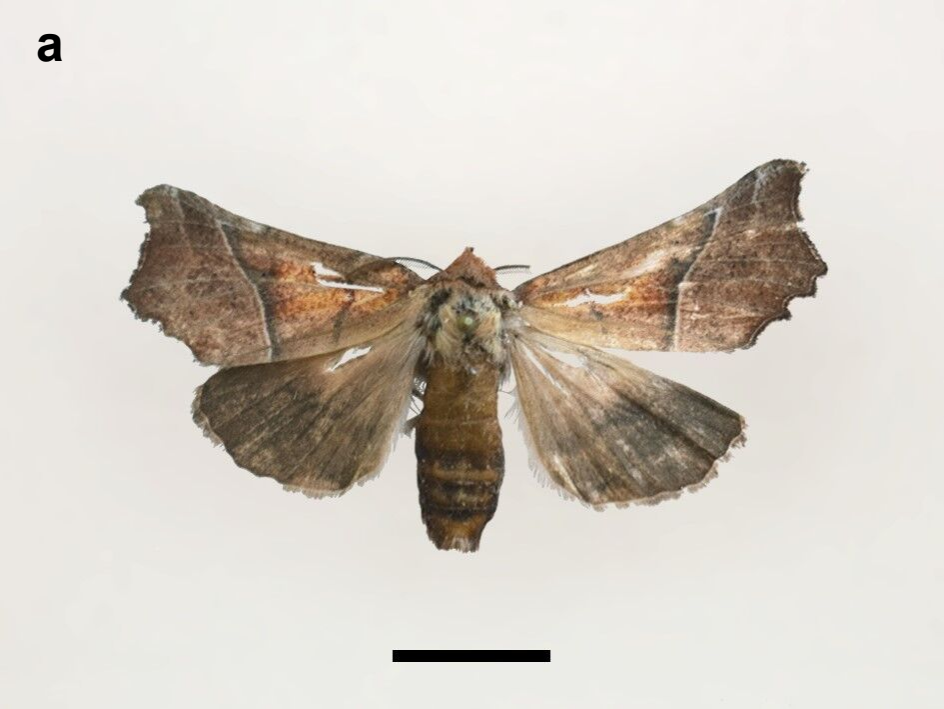
Male *Scoliopteryxlibatrix* (Linnaeus), Taddart, 13/06/2021;

**Figure 5b.**
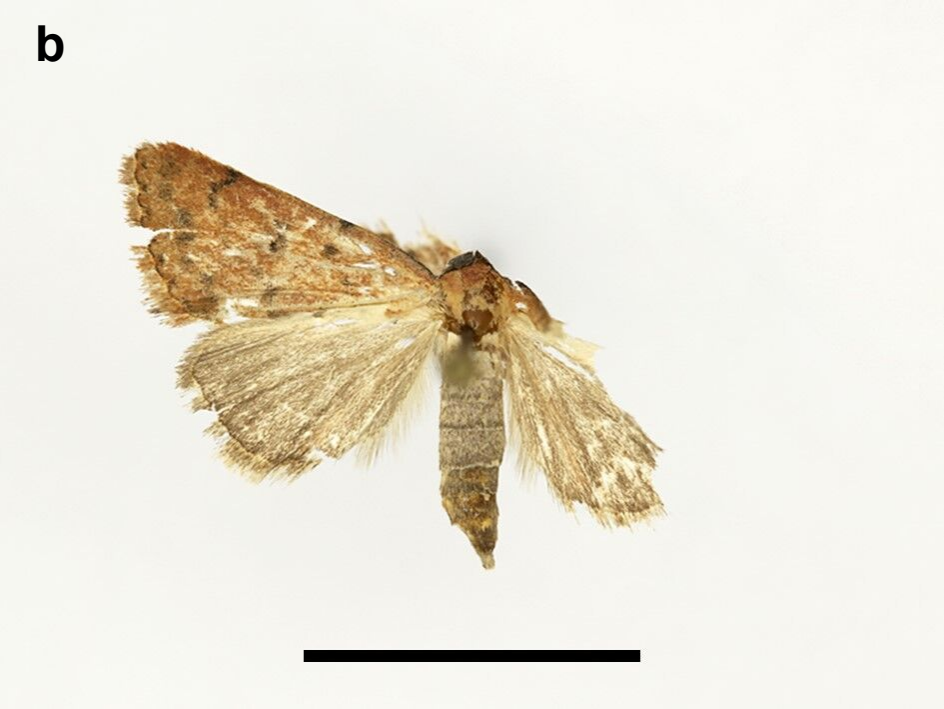
Male *Antharchaea* sp (near erubescens), Tassourte, 04/10/2022;

**Figure 5c.**
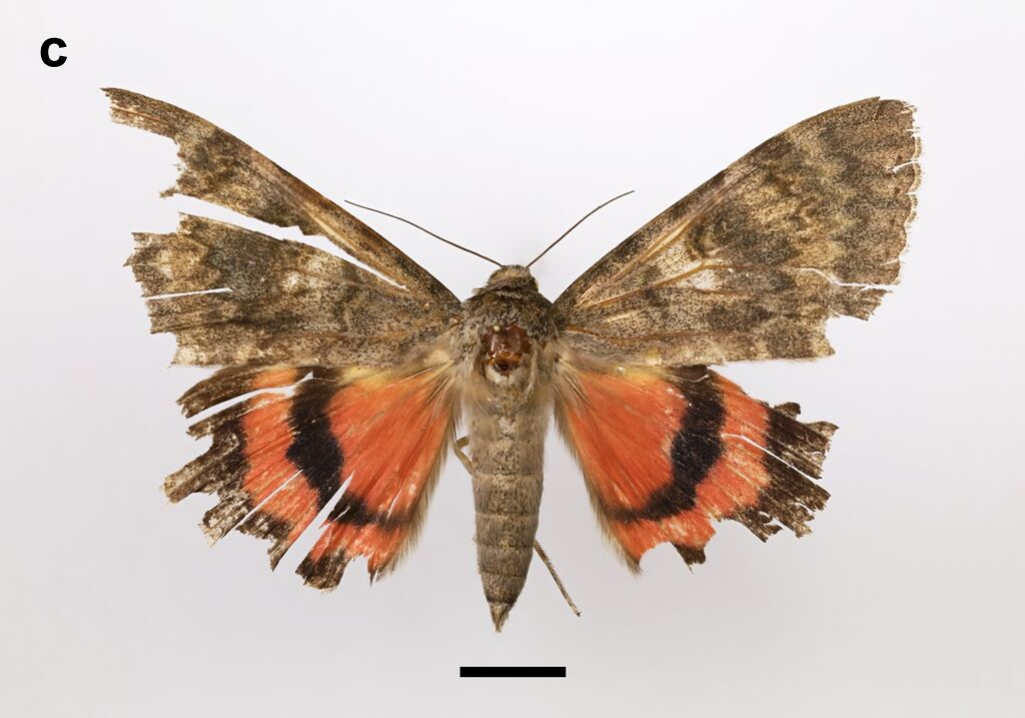
Male *Catocalaelocata* (Esper), Taddart, 02/10/2021;

**Figure 5d.**
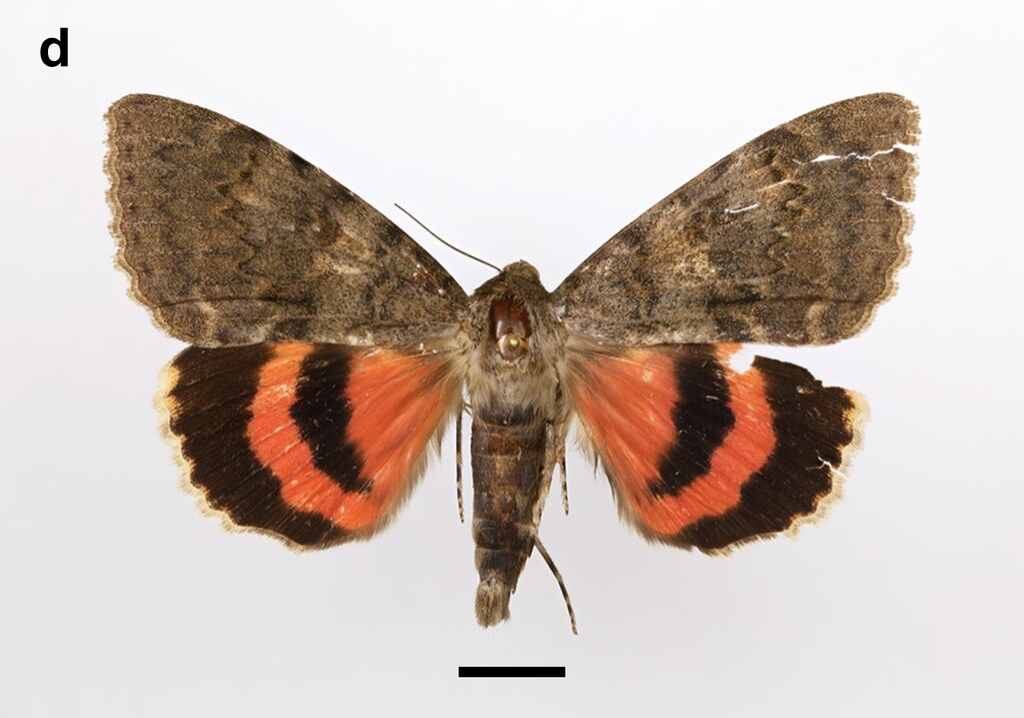
Male *Catocalaoberthuri* Austaut, Taddart, 04/07/2021;

**Figure 5e.**
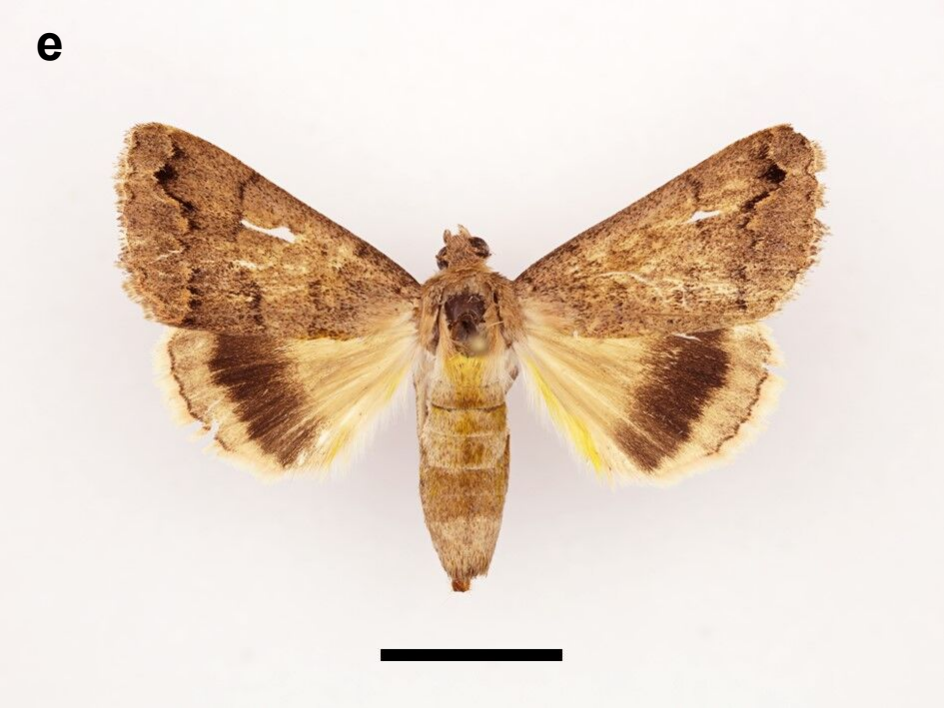
Female *Clytieillunaris* (Hübner), Ighalene, 23/09/2022;

**Figure 5f.**
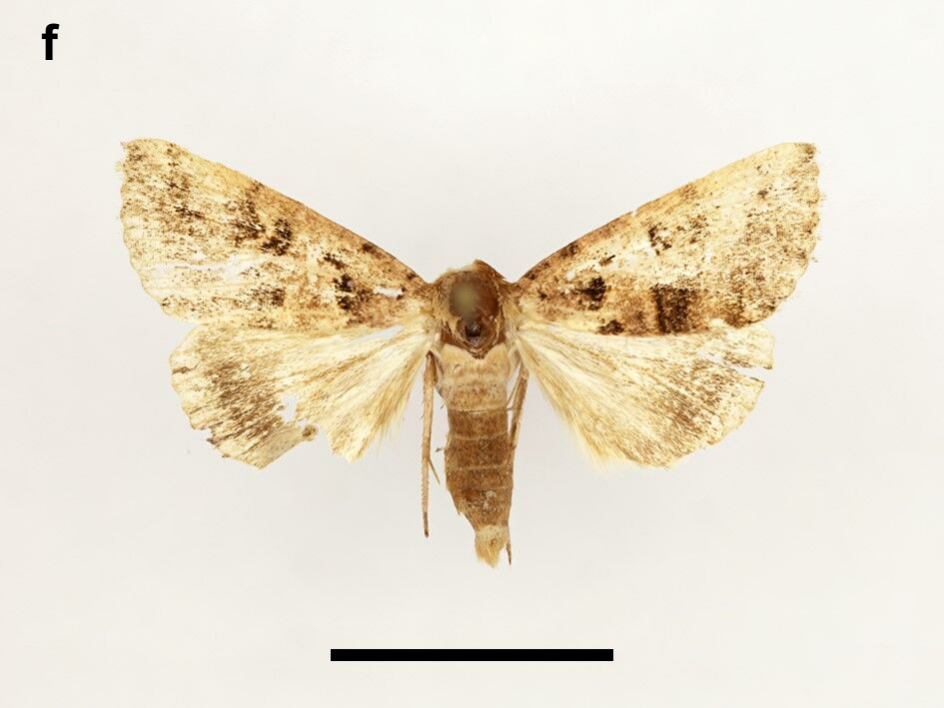
Male *Clytieinfrequens* (Swinhoe), Tamal , 22/09/2022.

**Figure 6a.**
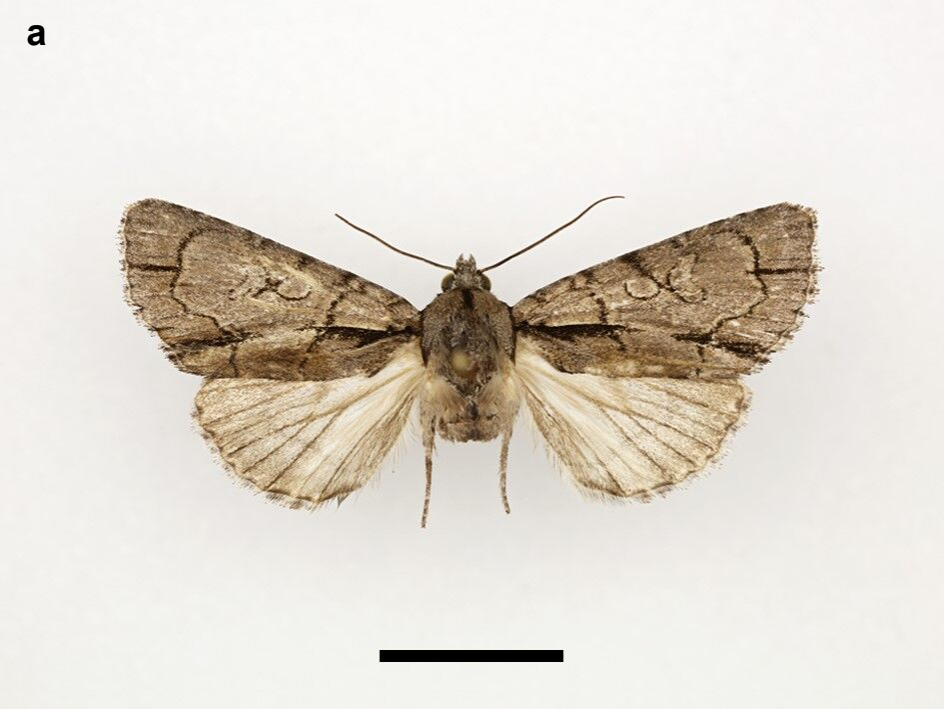
Habitus. Scale: 1 cm;

**Figure 6b.**
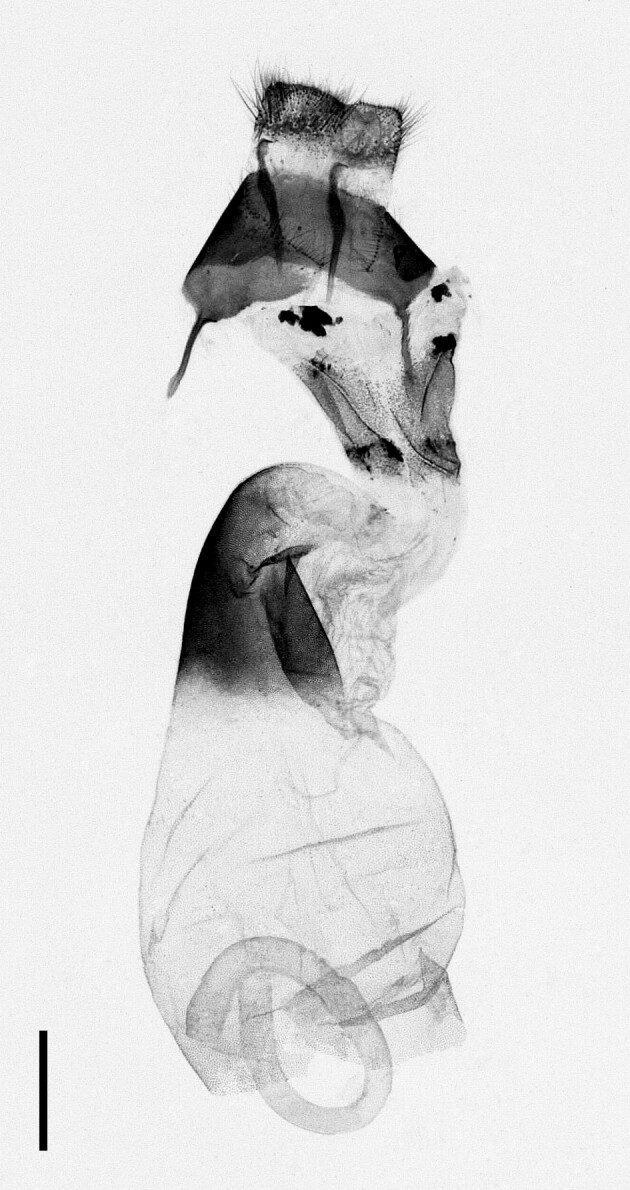
Genitalia. Scale: 1 mm.

**Figure 7a.**
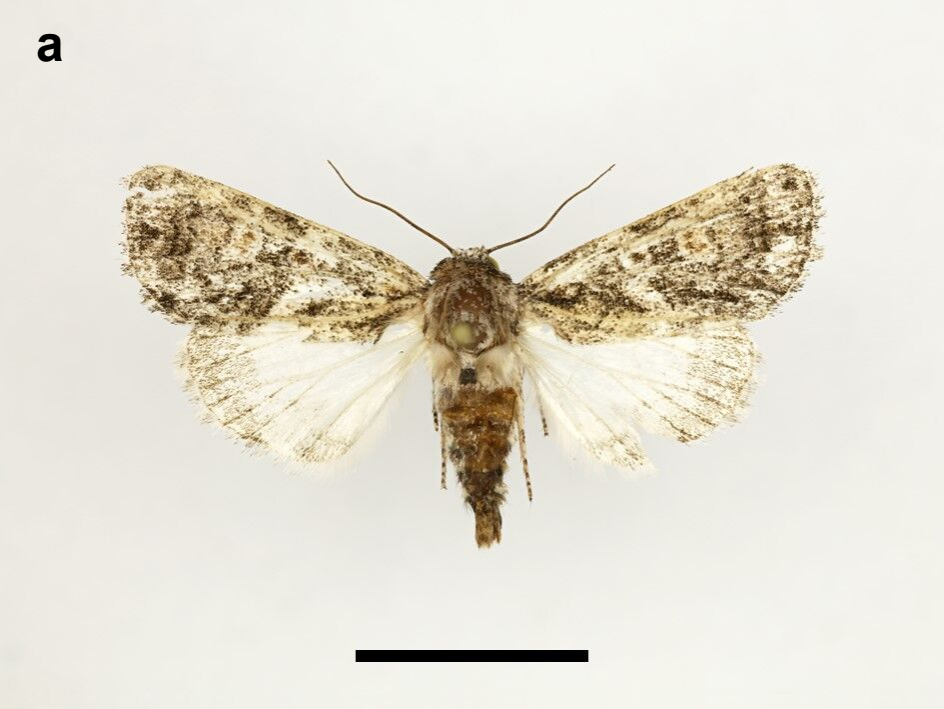
Female *Craniophorapontica* (Staudinger), Tamal, 04/10/2022;

**Figure 7b.**
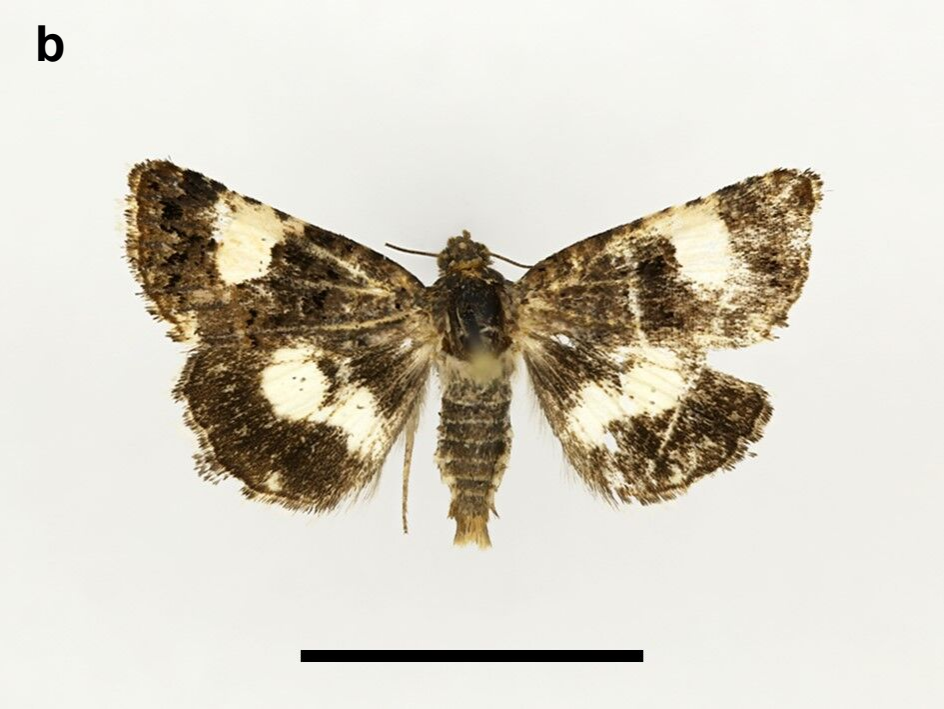
Female *Tytaluctuosa* (Denis & Schiffermüller), Timzilite, 04/10/2022.

**Figure 10a.**
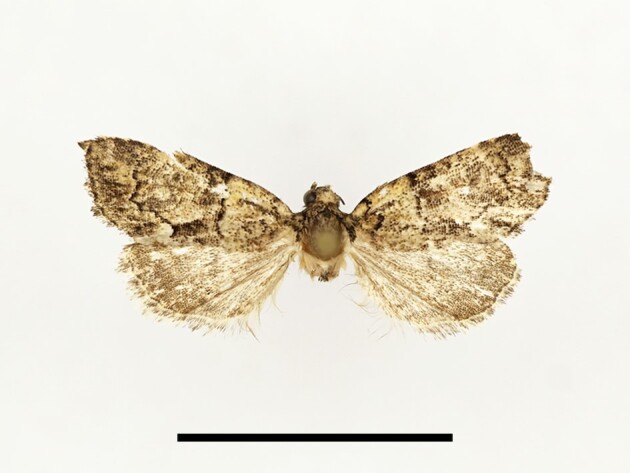
Habitus. Scale: 1 cm;

**Figure 10b.**
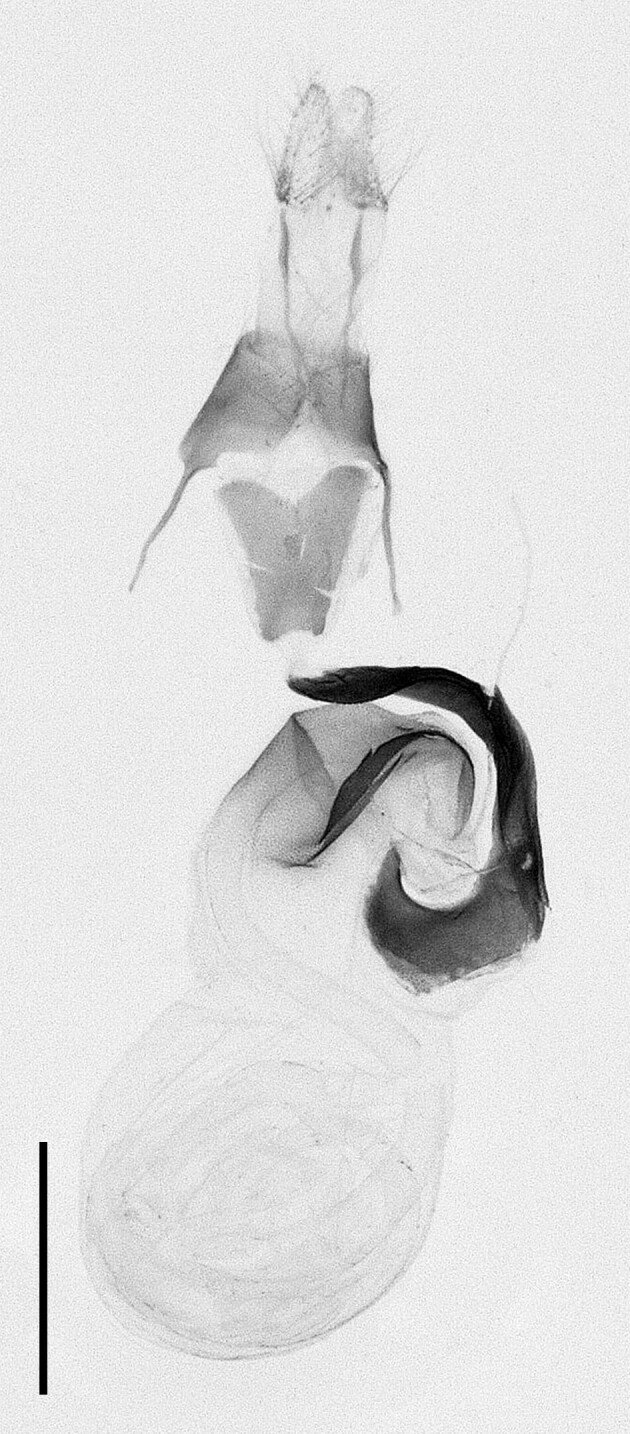
Genitalia. Scale: 1 mm.

**Figure 12a.**
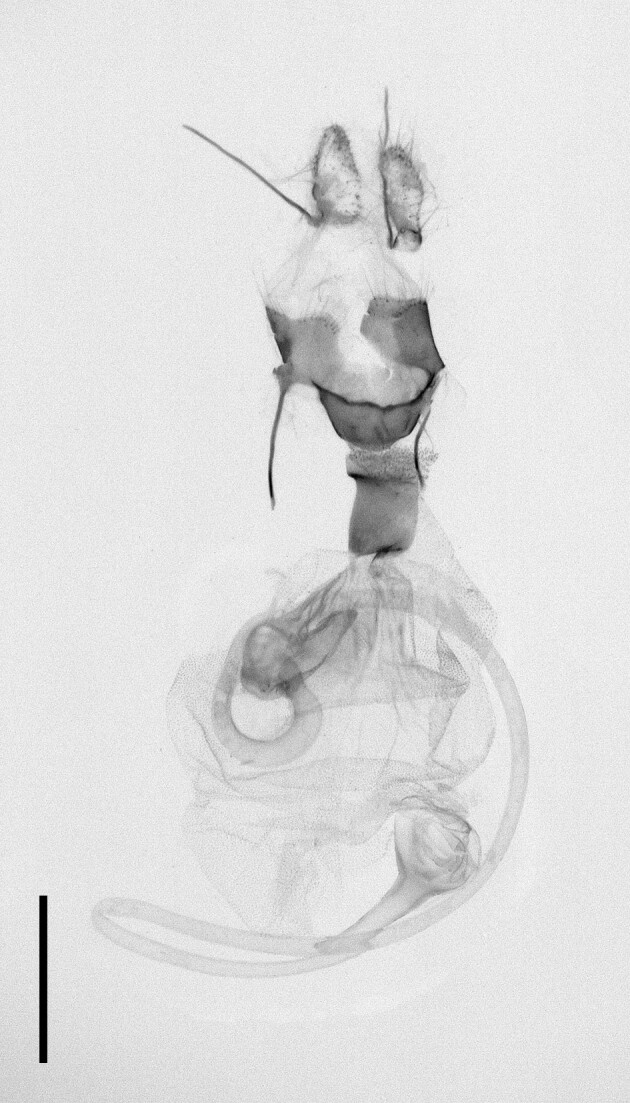
Female genitalia;

**Figure 12b.**
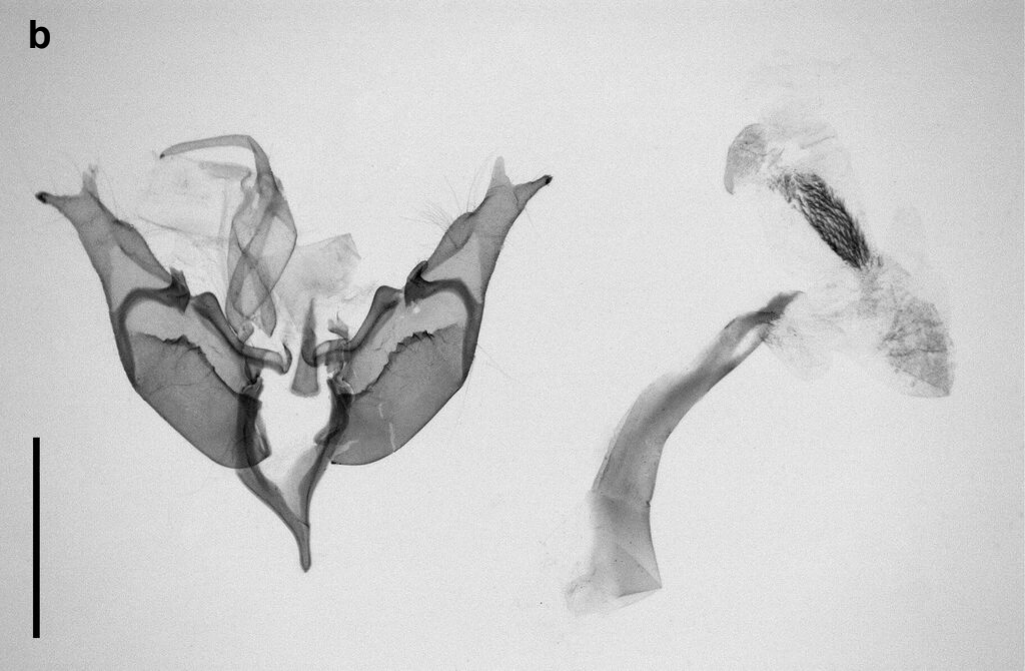
Male genitalia.

**Figure 13a.**
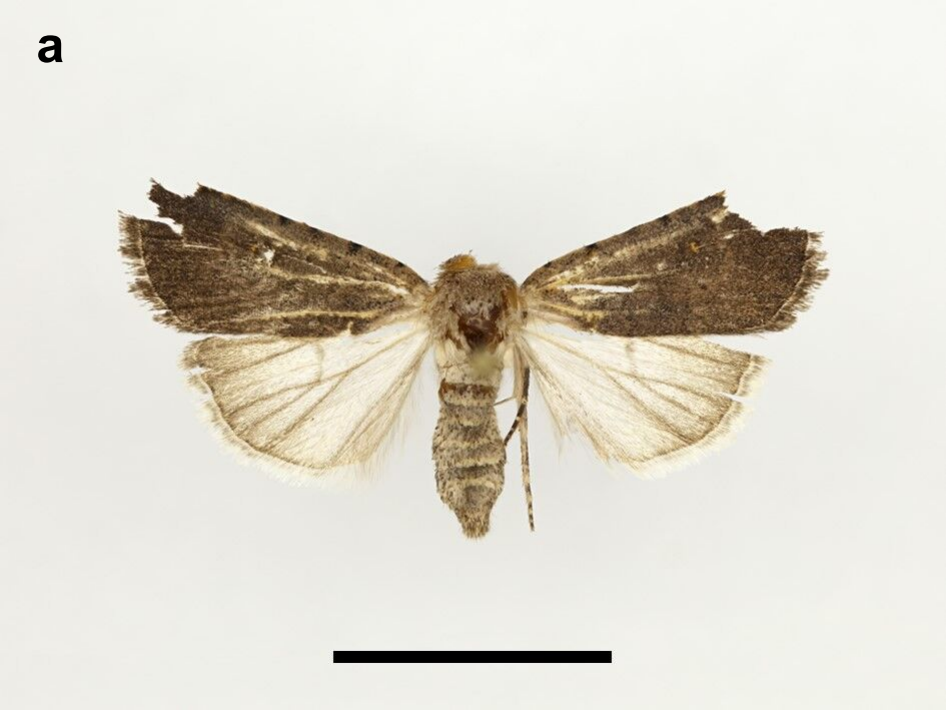
Female, taken at Tamal, 04/10/2022. Scale: 1 cm;

**Figure 13b.**
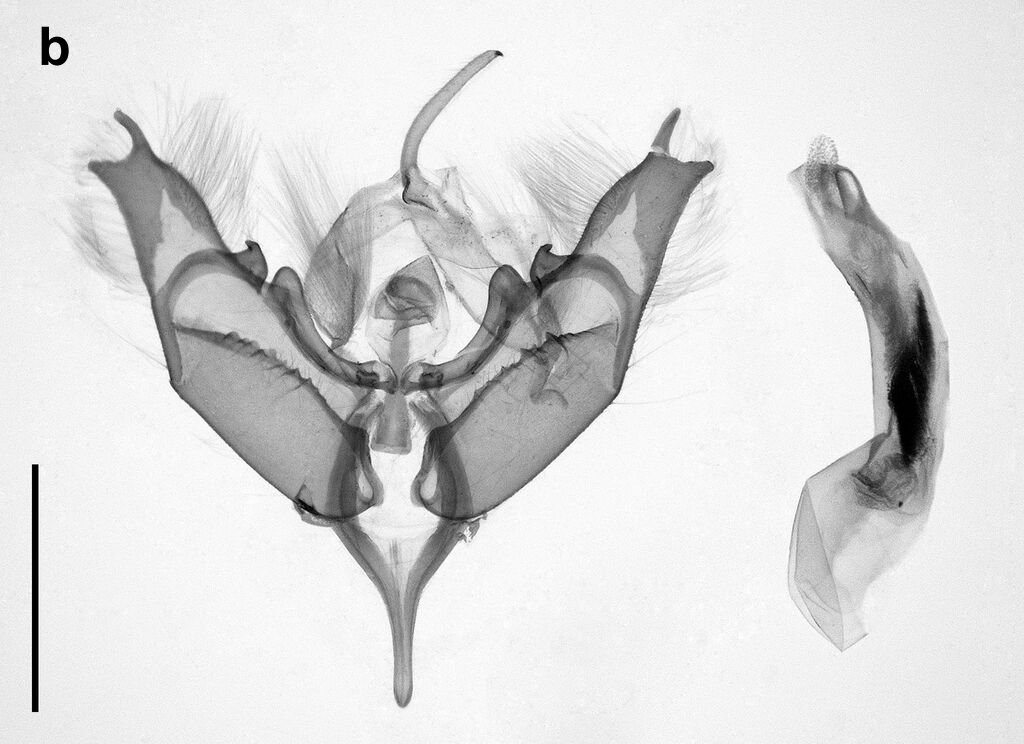
Genitalia of male, taken at Tamal, 04/10/2022. Scale: 1 mm.

**Figure 15a.**
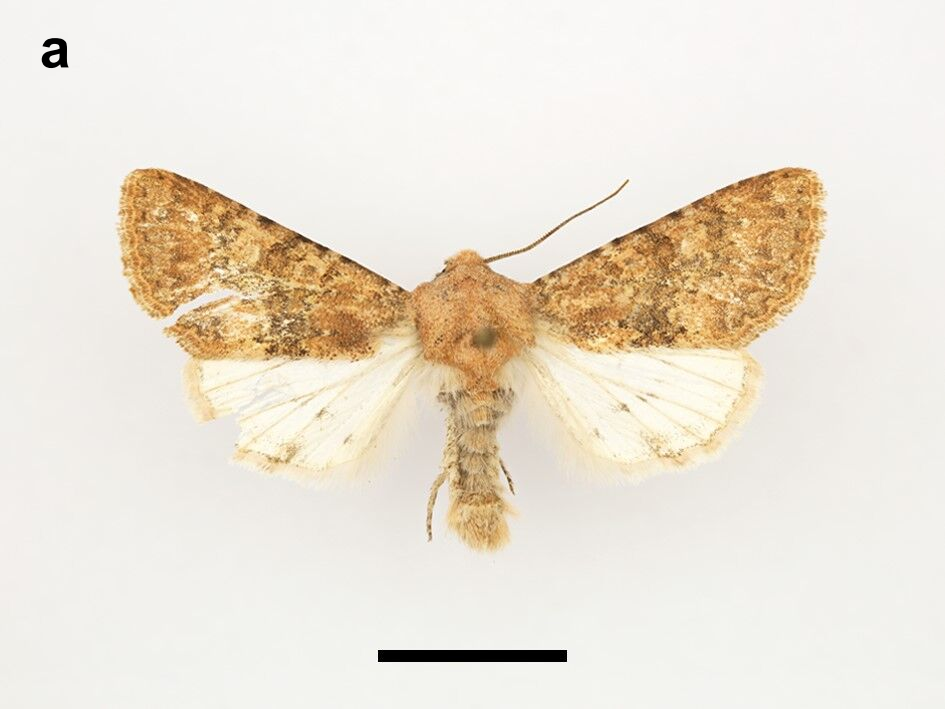
Male, taken at Tamal, 07/11/2022. Scale: 1 cm;

**Figure 15b.**
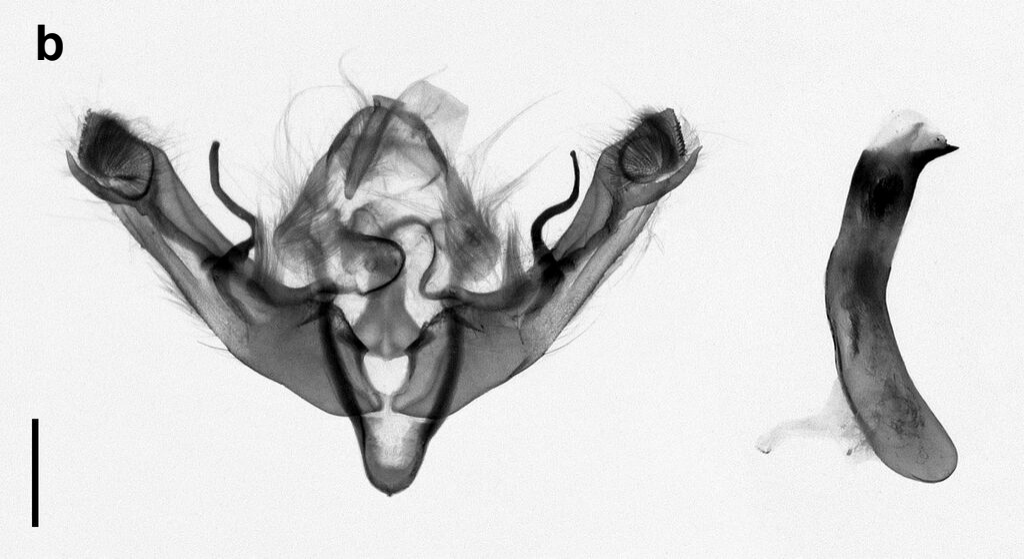
Genitalia of male, taken at Taddart, 02/10/2021. Scale: 1 mm.

**Figure 16a.**
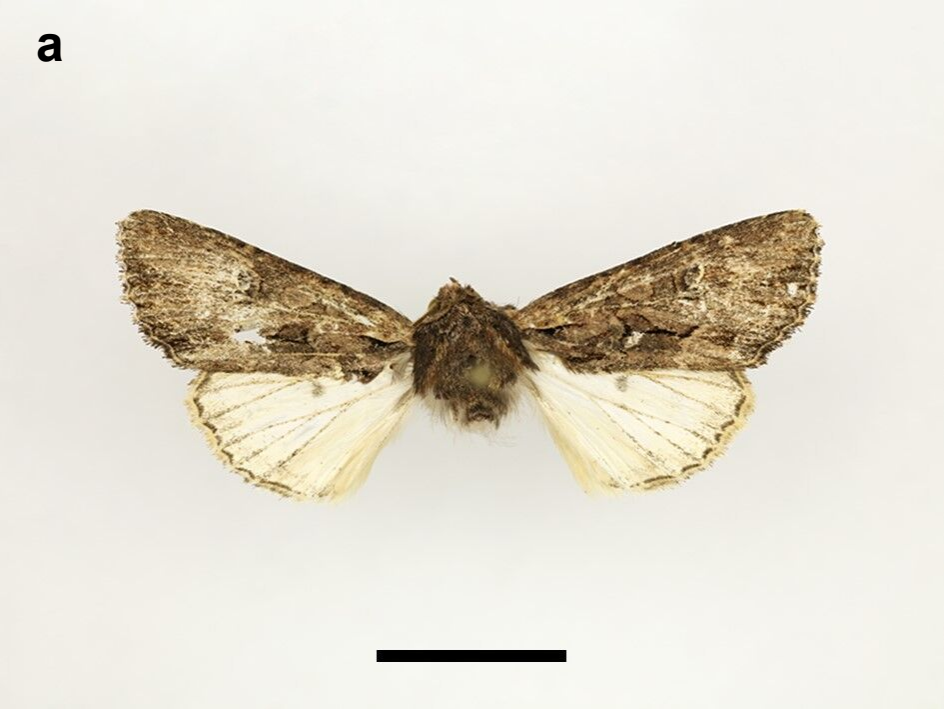
Male *Mniotypeoccidentalis* Yela, Fibiger, Ronkay & Zilli, Ighalene, 15/11/2022;

**Figure 16b.**
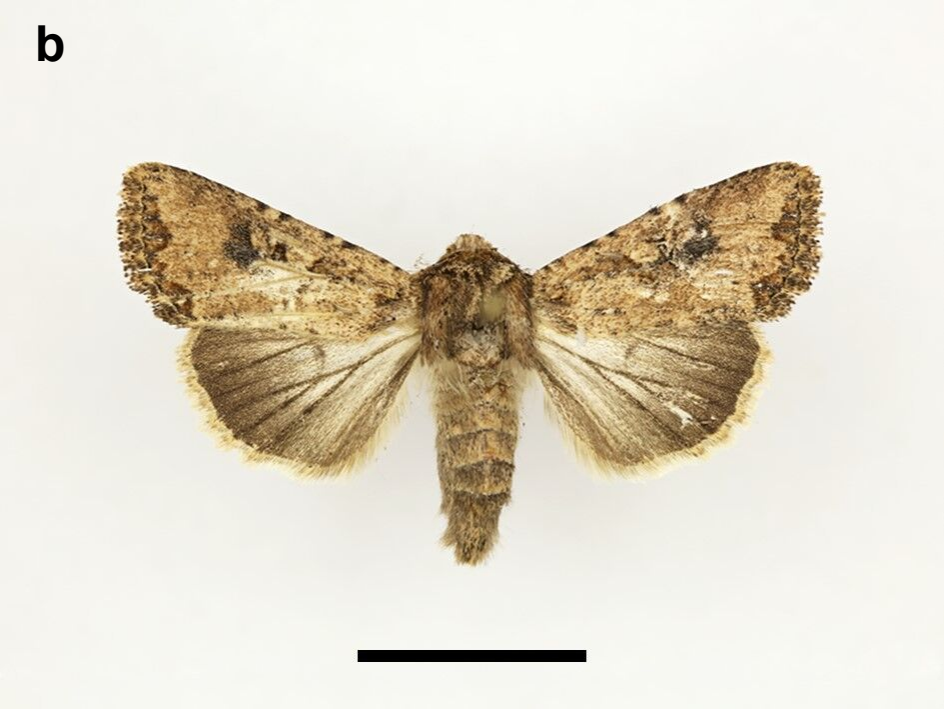
Female *Mythimnalanguida* (Walker), Timzilite, 15/11/2022;

**Figure 16c.**
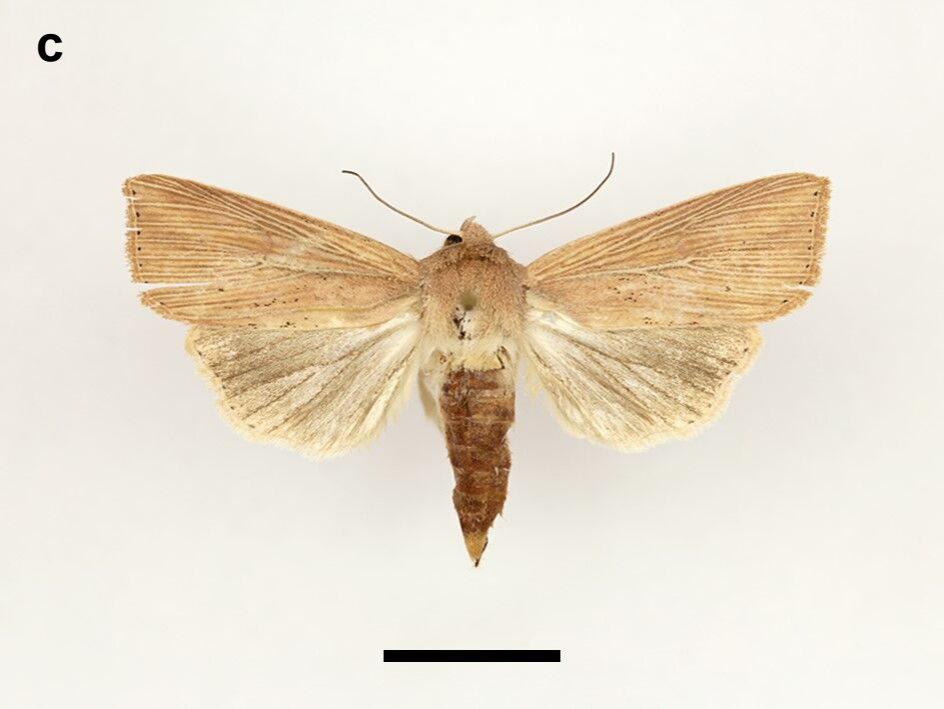
Female *Mythimnacongrua* (Hübner), Taddart, 18/09/2021;

**Figure 16d.**
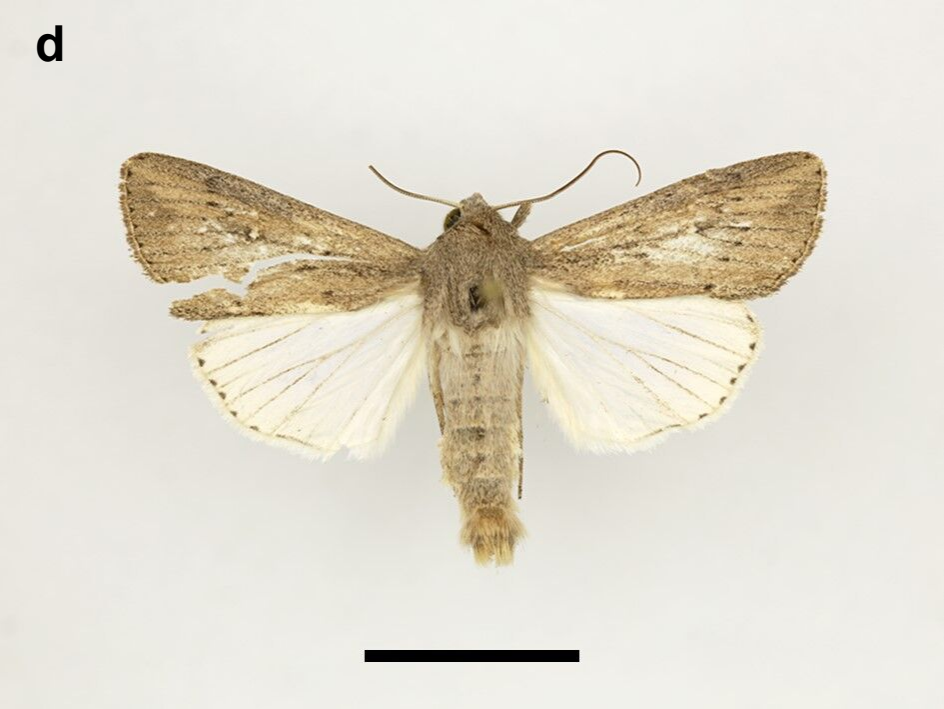
Male *Leucaniazeae* (Duponchel), Timzilite, 28/09/2022;

**Figure 16e.**
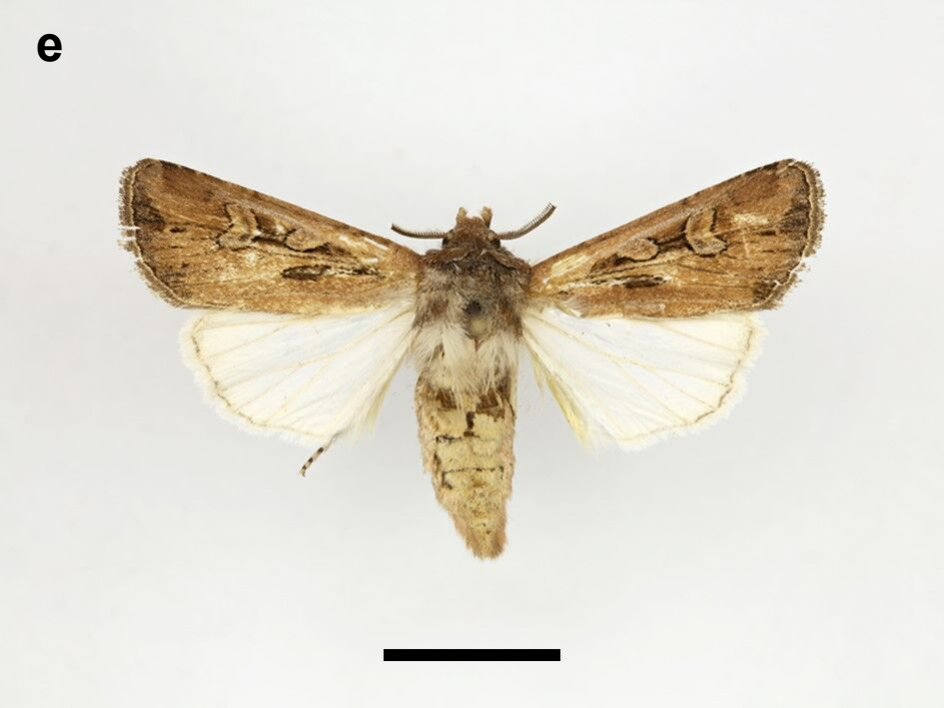
Male *Euxoatemera* (Hübner), Ait Wagestite, 27/09/2022;

**Figure 16f.**
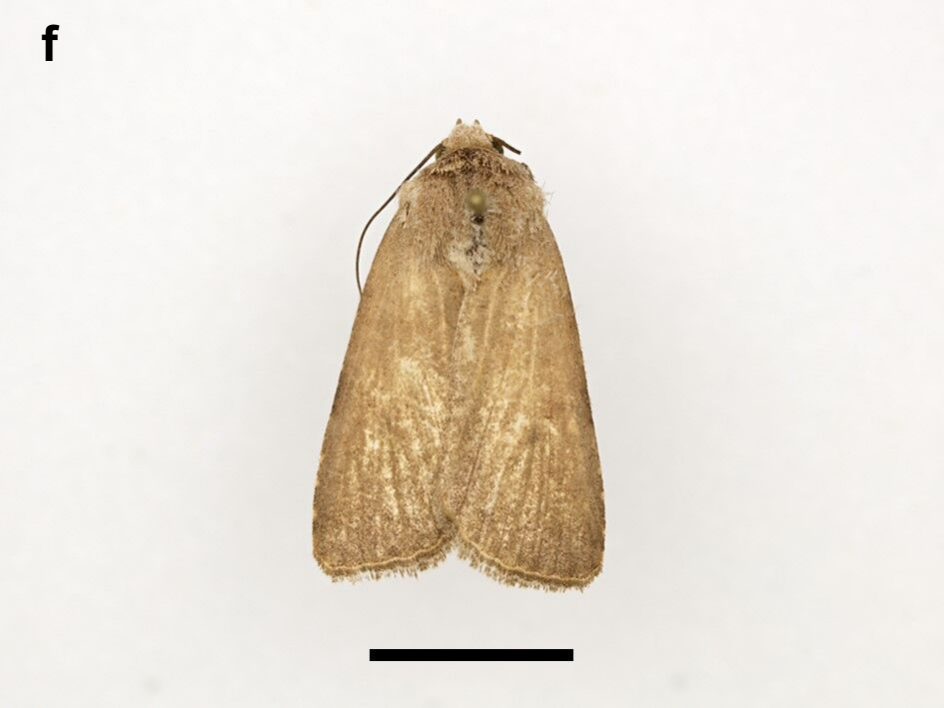
Female *Euxoacos* (Hübner), Taddart, 26/09/2021.

**Figure 17a.**
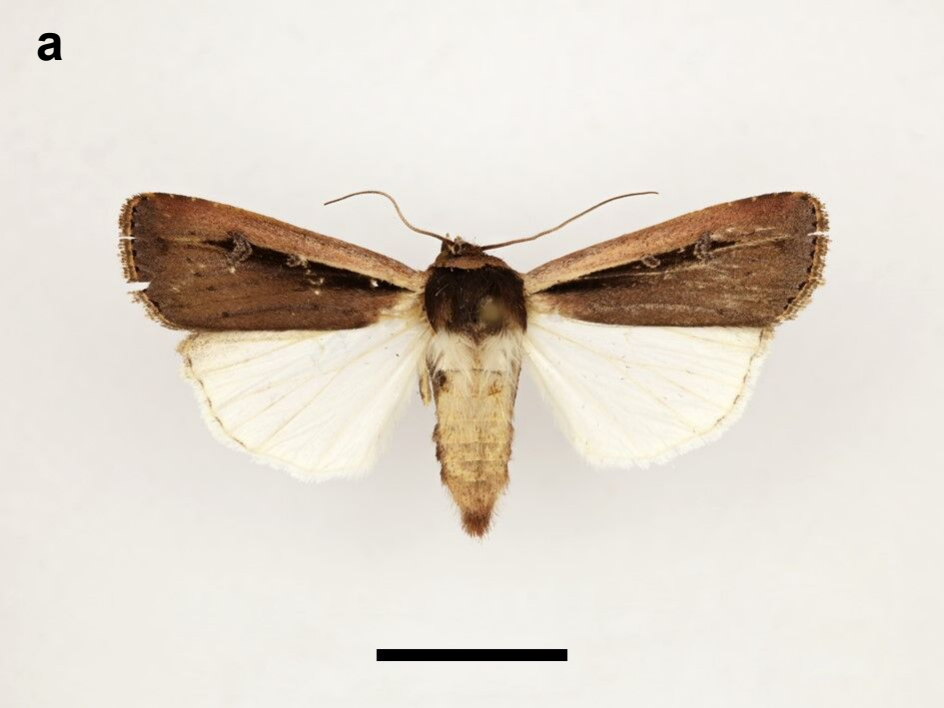
Female *Ochropleuraleucogaster* (Freyer), Timzilite, 28/09/2022;

**Figure 17b.**
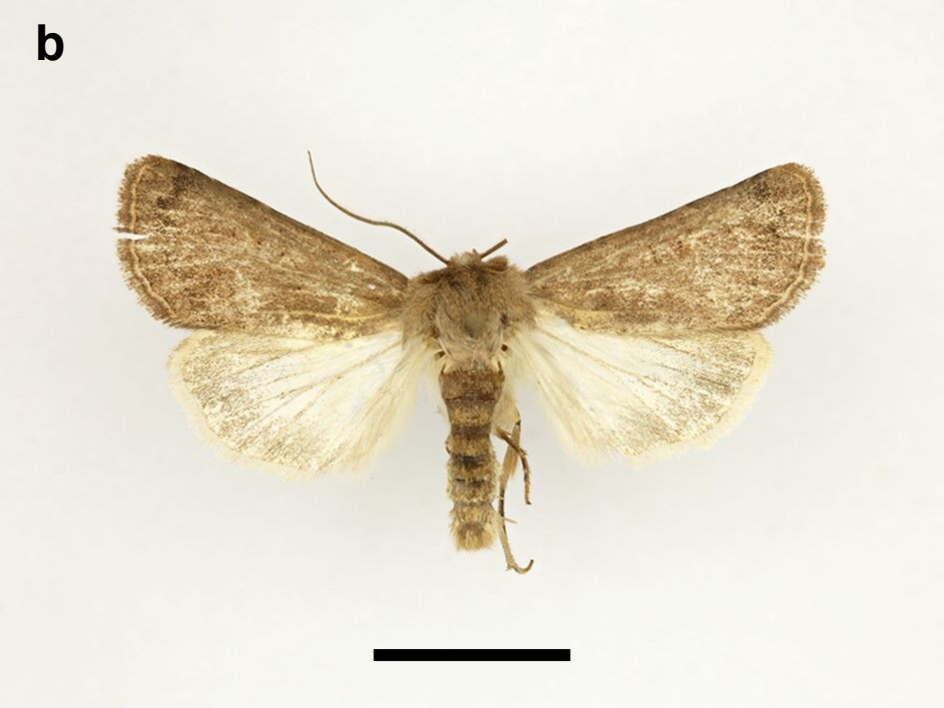
Male *Chersotisrungsi* Boursin, Taddart, 09/10/2021.

**Table 1.** Location of the study localities within the central High Atlas of Morocco.

**Locality**	**Latitude, Longitude**
Ait Ouiksane	31°24'55.10"N, 7°28'9.30"W
Ait Wagestite	31°23'19.40"N, 7°31'4.20"W
Azgour	31°20'57.40"N, 7°29'55.70"W
Ighalene	31°26'4.10"N, 7°31'43.00"W
Taddart	31°28'1.32"N, 7°20'11.78"W
Talatast	31°24'42.62"N, 7°31'14.80"W
Tamal	31°24'54.10"N, 7°32'29.00"W
Tassourte	31°27'38.30"N, 7°32'10.30"W
Timzilite	31°29'33.70"N, 7°32'8.90"W

## References

[B12062593] Arahou M (2008). Catalogue de l'entomofaune du Chêne vert du Moyen Atlas (Maroc). Documents de l’Institut Scientifique.

[B11996005] Audeoud G, Roch M (1938). Chasses printanières au lépidoptères au Maroc. Mitteilungen der Schweizerischen Entomologischen Gesellschaft.

[B11990080] Ayarza P., Alvarez-Lobato F., Teixell A., Arboleya M. L., Tesón E., Julivert M., Charroud M. (2005). Crustal structure under the central High Atlas Mountains (Morocco) from geological and gravity data. Tectonophysics.

[B11990318] Beauchamp W, Allmendinger R. W, Barazangi M, Demnati A, El Alji M, Dahmani M (1999). Inversion tectonics and the evolution of the High Atlas mountains, Morocco, based on a geological-geophysical transect. Tectonics.

[B11990092] Bouamri H., Boudhar A., Gascoin S., Kinnard C. (2018). Performance of temperature and radiation index models for point-scale snow water equivalent (SWE) simulations in the Moroccan High Atlas Mountains. Hydrological Sciences Journal.

[B11990329] Bouchaour-Djabeur S (2013). Les insectes ravageurs du Chêne liège au nord-ouest algérien (Devastating insects of Cork Oak in North-Western Algeria). Geo-Eco-Trop.

[B11990122] Boulamtat Rachid, Mesfioui Abdelhalim, El-Fakhouri Karim, Oubayoucef Ali, Sabraoui Abdelhadi, Aasfar Abderrahim, El-Bouhssini Mustapha (2021). Chemical composition, and insecticidal activities of four plant essential oils from Morocco against larvae of *Helicoverpaarmigera* (Hub.) under field and laboratory conditions. Crop Protection.

[B11990284] El Alami A, Fattah A, Bouzekraoui H (2021). Biodiversity, an essential component for the M’goun global geopark development (Morocco) - An overview. Journal of Analytical Sciences and Applied Biotechnology.

[B11990293] El Alami A (2022). Biodiversity loss in the Moroccan central High Atlas , its impact on local ecosystems and national economy , and wildlife conservation strategy : findings from 20 years of research. Journal of Analytical Sciences and Applied Biotechnology.

[B11995886] El Alaoui El Fels M. A, Roques A, Boumezzough A (1999). Les arthropodes liés aux galbules et aux graines du genévrier thurifère, Juniperus thurifera L., dans les Atlas marocains. Ecologia Mediterranea.

[B11995895] El Alaoui El Fels M. A, Yart A, Roques A, Arjouni Y., El Mercht S, Auger-Rozenberg M. A, Romane A (2013). Acariens et insectes ravageurs de deux cupressacées menacées au Maroc : le Genévrier thurifère et le cyprès de l’Atlas. Ecologia Mediterranea.

[B11990152] El Iraqui S., Hmimina M. (2016). Impact of Temperatures on the Voltinism of *Cydiapomonella* (Lepidoptera: Tortricidae). Annals of the Entomological Society of America.

[B12002244] Fetnassi N (2024). Moroccan moths specimens and observations.

[B11990355] Fibiger M (1990). Noctuinae I. Noctuidae Europeae.

[B11990363] Fibiger M (1993). Noctuinae II. Noctuidae Europaea.

[B11990371] Fibiger M (1997). Noctuinae III. Noctuidae Europaea.

[B11990379] Fibiger M, Hacker H (2007). Amphipyrinae, Condicinae, Eriopinae, Xyleninae. Noctuidae Europae.

[B11990387] Fibiger M, Ronkay L, Steiner A, Zilli A (2009). Pantheinae, Dilobinae, Acronictinae, Eustrotiinae, Nolinae, Bagisarinae, Acontiinae, Metoponiinae, Heliothinae and Bryophilinae. Noctuidae Europae.

[B11990395] Fibiger M, Ronkay L, Yela J. L, Zilli A (2010). Rivulinae, Boletobiinae, Hypenodinae, Araeopteroninae, Eublemminae, Herminiinae, Hypeninae, Phytometrinae, Euteliinae and Micronoctuidae, including Supplement to Noctuidae Europaeae.

[B12096245] Freitas A. V.L., Iserhard C. A, Santos J. P, Carreira J. Y.O, Ribeiro D. B, Melo D. H.A, Rosa A. H.B, Marini-Filho O. J, Accacio G. M, Uehara-Prado M (2014). Studies with butterfly bait traps: an overview. Revista Colombiana de Entomología.

[B11990403] Funnell D. C., Parish R. (1995). Environment and economic growth in the Atlas Mountains, Morocco: A policy-orientated research agenda. Mountain Research and Development.

[B11990412] Goater B, Ronkay L, Fibiger M (2003). Catocalinae and Plusiinae. Noctuidae Europaea.

[B11990428] Govi G, Fiumi G (2019). *Cryphiaclaudiae*, a new noctuid moth from Corsica. Quaderno Di Studi e Notizie Di Storia Naturale Della Romagna.

[B11990445] Grange J. C (2014). Première observation du migrateur *Pandesma robusta* (Walker, 1858) en France continentale (Lepidoptera, Noctuidae). https://r-a-r-e.fr/wp-content/uploads/2019/10/2014-XXIII-2.pdf.

[B11990454] Hacker H, Ronkay L, Fibiger M (2002). Hadeninae I. Noctuidae Europaea.

[B12060443] Hällfors Maria H, Heikkinen Risto K, Kuussaari Mikko, Lehikoinen Aleksi, Luoto Miska, Pöyry Juha, Virkkala Raimo, Saastamoinen Marjo, Kujala Heini (2023). Recent range shifts of moths, butterflies, and birds are driven by the breadth of their climatic niche. Evolution Letters.

[B11990170] Hatami Marzieh, Ziaee Masumeh, Seraj Ali Asghar, Mehrabi-Koushki Mehdi, Francikowski Jacek (2021). Effects of imunit insecticide on biological characteristics and life table parameters of *Spodopteracilium* (Lepidoptera: Noctuidae). Insects.

[B11995856] Hausmann A (1997). Interessante nordafrikanische Geometridenarten aus der Sammlung Herbulot, Paris (Lepidoptera, Geometridae). facetta - Berichte der Entomologischen Gesellschaft Ingolstadt e.V..

[B11990479] Hausmann A, Leipnitz M, Blaesius R (2008). *Idaeaomari* Hausmann & Blasius, sp. n. from Morocco (Lepidoptera: Geometridae, Sterrhinae). SHILAP Revista de Lepidopterologia.

[B11990189] Hebert Paul D. N., Penton Erin H., Burns John M., Janzen Daniel H., Hallwachs Winnie (2004). Ten species in one: DNA barcoding reveals cryptic species in the neotropical skipper butterfly *Astraptesfulgerator*. Proceedings of the National Academy of Sciences.

[B11990488] Hreblay M (1994). New taxa of the tribe Orthosiini, 4. (Lepidoptera, Noctuidae). Zoologica Academiae Scientiarum Hungaricae.

[B11990199] Husemann Martin, Schmitt Thomas, Zachos Frank E., Ulrich Werner, Habel Jan Christian (2013). Palaearctic biogeography revisited: evidence for the existence of a North African refugium for Western Palaearctic biota. Journal of Biogeography.

[B11990209] Kacha S, Adamou-Djerbaoui M, Marniche F, De Prins W (2017). The richness and diversity of Lepidoptera species in different habitats of the national Park Theniet El Had (Algeria). Journal of Fundamental and Applied Sciences.

[B11990498] Kaila L, Mutanen M, Sihvonen P, Tyllinen J, Tabell J (2019). Characterization of Pleurotinae, with review of *Pleurota* species close to *P.aristella* (Linnaeus) from Morocco (Lepidoptera: Gelechioidea: Oecophoridae). Zootaxa.

[B11996046] Katbeh-Bader A. (2017). Contribution to the Erebidae of Jordan (Lepidoptera: Erebidae). SHILAP Revista de lepidopterología.

[B11990509] Koçak A. Ö, Kemal M (2009). Third report on the temporary results of the lepidopteran list of Africa continent based upon the info-system of the Cesa. Cesa Publications on African Lepidoptera (Royal Museum for Central Africa).

[B11990218] Laaksonen Jesse, Laaksonen Toni, Itämies Juhani, Rytkönen Seppo, Välimäki Panu (2006). A new efficient bait-trap model for Lepidoptera surveys – the “Oulu” model. Entomologica Fennica.

[B11996014] Le Cerf F (1924). Lépidoptères hétérocères nouveau du Maroc. Bulletin de la Société entomologique de France.

[B11990518] Leraut P (2009). Geometrid Moths. Moths of Europe.

[B11990526] Leraut P (2019). Noctuids 1. Moths of Europe.

[B11990547] Leraut P (2019). Noctuids 2. Moths of Europe.

[B11990228] Médail Frédéric, Quézel Pierre (2001). Biodiversity hotspots in the Mediterranean basin: Setting global conservation priorities. Conservation Biology.

[B11995042] Mérit X (2014). [Lepidopterological observations in the Moroccan Anti-Atlas (April 2011) (Lepidoptera: Rhopalocera and Heterocera).]. Lepidopteres (Paris).

[B11995848] Mironov V (2003). Larentiinae II (Perizomini and Eupitheciini). - In A. Hausmann (ed.): The Geometrid Moths of Europe.

[B11995142] Mokhles A (1989). Contribution a la connaissance des Lépidopteres du Maroc. Observations entomologiques en automne a Azrou (Moyen-Atlas 1.250-1.500m.). SHILAP Revista de Lepidopterologia.

[B11995159] Mokhles A (1995). Note brève de captures nocturnes fin aout 1990 en Moulouya - Maroc oriental. Cercle Des Lepidopteristes de Belgique Bulletin.

[B11995168] Mokhles A (1996). [10th contribution to the knowledge of Lepidoptera from the Moroccan Atlas mountains, visit in June 1992.]. Cercle Des Lepidopteristes de Belgique Bulletin.

[B11995926] Mostakim L, Guennoun F. Z, Fetnassi N, Ghamizi M (2022). Analysis of floristic diversity of the forest ecosystems of the Zat valley- High Atlas of Morocco: Valorization and Conservation perspectives. Journal of Advanced Biotechnology and Experimental Therapeutics.

[B11990237] Myers N (2003). Biodiversity hotspots revisited. BioScience.

[B11990246] Õunap E, Choi S. E, Matov A, Tammaru T (2021). Description of *Nolaestonica* sp. nov., with comparison to *N.aerugula* and *N.atomosa* stat. rev. (Lepidoptera, Nolidae, Nolinae). Zootaxa.

[B12096236] Pettersson L. B, Franzén M (2008). Comparing wine-based and beer-based baits for moth trapping: a field experiment. Entomologisk Tidskrift.

[B11990255] Rankou Hassan, Culham Alastair, Sghir Taleb Mohammed, Ouhammou Ahmed, Martin Gary, Jury Stephen L. (2015). Conservation assessments and Red Listing of the endemic Moroccan flora (monocotyledons). Botanical Journal of the Linnean Society.

[B11995185] Ratzel U (2018). Zur Kenntnis der Blütenspanner (*Eupithecia* Curtis, 1825) Marokkos mit Beschreibung einer neuen Unterart (Lepidoptera: Geometridae, Larentiinae, Eupitheciini). Entomologische Zeitschrift-Schwanfeld.

[B11996074] Rezbanyai-Reser L, Hausmann A (2000). Über Mythimna (Morphopoliana) languida (Walker, 1858), eine neue, tropische Wanderfalterart Europas, und ihre Fundangaben in Nord- und Süditalien (Lepidoptera: Noctuidae). Atalanta.

[B11996083] Rezbanyai-Reser L, Hausmann A (2000). Eine Berichtigung: Mythimna (Morphopoliana) languida (Walker, 1858) auch in Deutschland und Makedonien. Atalanta.

[B11995660] Ronkay G, Ronkay L, Gyulai P (2011). A Taxonomic Atlas of the Eurasian and North African Noctuoidea. Cucullinae II and Psaphidinae. – The Witt Catalogue.

[B11995652] Ronkay L, Yela J. L, Hreblay M (2001). Hadeninae II. Noctuidae Europae.

[B11996023] Rungs E. E. C (1938). Notes de Lépidoterologie marocaine. III: Addition à la faune des Lépidoptères Noctuidae du Maroc. Bulletin de la Société des Sciences naturelles du Maroc.

[B11995668] Rungs E. E. C (1979). Catalogue raisonné des Lépidoptères du Maroc.

[B11995676] Rungs E. E. C (1981). Catalogue raisonnée des Lépidoptères du Maroc.

[B11990266] Salem Abdelfattah, (2021). Revision of Lepidoptera of Egypt, Superfamily Noctuoidea Part II: Erebidae, Nolidae and Euteliidae. Egyptian Academic Journal of Biological Sciences. A, Entomology.

[B11990275] Sbay Hassan, Zas Rafael (2018). Geographic variation in growth, survival, and susceptibility to the processionary moth (*Thaumetopoeapityocampa* Dennis & Schiff.) of *Pinushalepensis* Mill. and *P.brutia* Ten.: results from common gardens in Morocco. Annals of Forest Science.

[B12096186] Süssenbach D, Fiedler K (1999). Noctuid moths attracted to fruit baits: Testing models and methods of estimating species diversity. Nota Lepidopterologica.

[B11995684] Tabell J, Mutanen M, Sihvonen Pasi (2019). Three morphologically and genetically confirmed new *Pleurota* species from Morocco (Lepidoptera: Gelechioidea: Oecophoridae: Pleurotinae). Zootaxa.

[B11995693] Tarrier M. R, Declare J (2008). Les Papillons de jour du Maroc: Guide d’identification et de bioindication.

[B11995701] Tarrier M. R, Andre J. M (2016). Le Maroc revisité... suite et fin (Premiere partie) (Lepidoptera
Papilionoidea et Zygaenidae
Zygaeninae). Alexanor.

[B11995710] Tarrier M. R, Andre J. M (2017). Le Maroc revisité… suite et fin (Troisième partie) (Lepidoptera
Papilionoidea et Zygaenidae
Zygaeninae). Alexanor.

[B11995728] Tarrier M. R (2018). Le Maroc revisité… suite et fin (Addenda) (Lépidoptera Papilionoidea). Alexanor.

[B11995719] Tarrier M. R, Andre J. M (2018). Le Maroc revisité… suite et fin (Quatrième partie) (Lépidoptera Papilionoidea et Zygaenidae
Zygaeninae). Alexanor.

[B11995737] Teobaldelli A (1987). Voyage entomologique au Maroc. Cercle Des Lepidopteristes de Belgique Bulletin.

[B11995829] Tiberi R, Branco M, Bracalini M, Croci F, Panzavolta T (2016). Cork oak pests: a review of insect damage and management. Annals of Forest Science.

[B12064009] Top M, Fritsch D, Kononenko V (2023). Noctuidae Europaeae Essential.

[B12095355] Utrio P (1983). Sugaring for moths: why are noctuids attracted more than geometrids?. Ecological Entomology.

[B11995763] Varga Z, Gyulai P, Ronkay L, Ronkay G (2013). A Taxonomic Atlas of the Eurasian and North African Noctuoidea. Noctuinae I. – The Witt Catalogue.

[B12494317] Walker J (1890). Notes on the Lepidoptera from the region of the Straits of Gibraltar.. Transactions of the Entomological Society of London.

[B12060459] Warren M. S., Hill J. K., Thomas J. A., Asher J., Fox R., Huntley B., Roy D. B., Telfer M. G., Jeffcoate S., Harding P., Jeffcoate G., Willis S. G., Greatorex-Davies J. N., Moss D., Thomas C. D. (2001). Rapid responses of British butterflies to opposing forces of climate and habitat change. Nature.

[B11995791] Yela J. L, De Vrieze M (2002). *Mythimna* (Morphopoliana) *languida* (Walker, 1858): first records for the Ibero-balearic area (Lepidoptera: Noctuidae: Hadeninae). Boletín S.E.A.

[B11995821] Zilli A, Varga Z, Ronkay G, Ronkay L (2009). A Taxonomic Atlas of the Eurasian and North African Noctuoidea. Apameini I. – The Witt Catalogue.

